# Folate Deficiency Triggered Apoptosis of Synoviocytes: Role of Overproduction of Reactive Oxygen Species Generated via NADPH Oxidase/Mitochondrial Complex II and Calcium Perturbation

**DOI:** 10.1371/journal.pone.0146440

**Published:** 2016-01-15

**Authors:** Hung-Chih Hsu, Wen-Ming Chang, Jin-Yi Wu, Chin-Chin Huang, Fung-Jou Lu, Yi-Wen Chuang, Pey-Jium Chang, Kai-Hua Chen, Chang-Zern Hong, Rang-Hui Yeh, Tsan-Zon Liu, Ching-Hsein Chen

**Affiliations:** 1 Department of Physical Medicine and Rehabilitation, Chia-Yi Chang Gung Memorial Hospital, Chia-Yi, Taiwan; 2 Department of Nursing, Chang-Gung University of Science and Technology, Chia-Yi, Taiwan; 3 Graduate Institute of Clinical Medical Sciences, College of Medicine, Chang Gung University, Taoyuan, Taiwan; 4 Center of Advanced Integrative Sports Medicine, Chia-Yi Chang Gung Memorial Hospital, Chia-Yi, Taiwan; 5 Department of Microbiology, Immunology and Biopharmaceuticals, Collage of Life Sciences, National Chiayi University, Chiayi City 60004, Taiwan; 6 Institute of Medicine, Chung Shan Medical University, Taichung, Taiwan; 7 Department of Physical therapy, Hung Kuang University, Taichung, Taiwan; 8 Translational Research Laboratory, Cancer Center, Taipei Medical University and Hospital, Taipei, Taiwan; School of Medicine and Health Sciences, University of North Dakota, UNITED STATES

## Abstract

Despite a plethora of literature has documented that osteoarthritis (OA) is veritably associated with oxidative stress-mediated chondrocyte death and matrix degradation, yet the possible involvement of synoviocyte abnormality as causative factor of OA has not been thoroughly investigated. For this reason, we conduct the current studies to insight into how synoviocytes could respond to an episode of folate-deprived (FD) condition. First, when HIG-82 synoviocytes were cultivated under FD condition, a time-dependent growth impediment was observed and the demise of these cells was demonstrated to be apoptotic in nature mediated through FD-evoked overproduction of reactive oxygen species (ROS) and drastically released of cytosolic calcium (Ca^2+^) concentrations. Next, we uncovered that FD-evoked ROS overproduction could only be strongly suppressed by either mitochondrial complex II inhibitors (TTFA and carboxin) or NADPH oxidase (NOX) inhibitors (AEBSF and apocynin), but not by mitochondrial complex I inhibitor (rotenone) and mitochondrial complex III inhibitor (antimycin A). Interestingly, this selective inhibition of FD-evoked ROS by mitochondrial complex II and NOX inhibitors was found to correlate excellently with the suppression of cytosolic Ca^2+^ release and reduced the magnitude of the apoptotic TUNEL-positive cells. Taken together, we present the first evidence here that FD-triggered ROS overproduction in synoviocytes is originated from mitochondrial complex II and NOX. Both elevated ROS in tandem with cytosolic Ca^2+^ overload serve as final arbitrators for apoptotic lethality of synoviocytes cultivated under FD condition. Thus, folate supplementation may be beneficial to patients with OA.

## Introduction

Osteoarthritis (OA) is a time- and age-dependent process leading to aberrant cartilage structure which is characterized by decreased number of chondrocytes, deterioration of existing cartilage extracellular matrix, and abnormality in composition and pathologic matrix calcification [1].

Cellular redox homeostasis is maintained by the balance between reactive oxygen species (ROS) generation and elimination. However, when this balance is tilted in favor of the state of increased ROS generation is referred to as oxidative stress. Despite oxidative stress has been incriminated as causative factor in the pathogenesis of OA [2,3], yet, the involvement of synoviocyte functional abnormality as a possible contributing factor of OA has not previously been investigated.

Folic acid (folate; vitamin B9) is an essential micronutrient which serves as critical coenzymes for purine and thymidylate biosynthesis and biological methylation of macromolecules and remethylation of homocysteine (Hcy) back to methionine [4,5,6]. A plethora of literature has documented that folate deficiency (FD) could trigger ROS overproduction and intracellular calcium overloading leading to the occurrence of apoptosis in many cell types [7,8,9,10,11]. In addition, FD-instigated oxdative stress has been directly or indirectly involved in the pathogenesis of many diseases such as cardiovascular diseases, anemia, fetus neural tube defect, cancer, Alzheimer’s disease [7,12,13,14,15,16,17,18,19]. Thus, FD-induced oxidative stress could be constituted as one of the risk factors for a variety of diseases.

FD may occur at all ages, particularly in persons ingesting a poor diet or suffering from intestinal malabsorption or who have excessive alcohol intake [20]. Clarke et al. [21] reported that the prevalence of FD increase with age which was correlated excellently with the occurrence of OA in the elderly population, which will create a major health care challenge and places an enormous economic burden on society [22].

FD can predominantly trigger oxidative stress-mediated accumulation of Hcy, the latter is known to modulate bone remodeling through several known mechanisms such as increasing in osteoclast activity in tandem with decreasing osteoclast function and direct action of Hcy on bone matrix. These observed effects were demonstrated to be ascribable to the activation of metalloproteinases (MMPs) that degrade extracellular bone matrix [23]. Furthermore, the underlying mechanism associated with this observed phenomenon was probably attributed to the activation of NF-κB via Hcy-instigated H_2_O_2_ production as analogous to the literature reported elsewhere [24]. Along the same vein, FD was previously demonstrated to activate inducible NO synthase (iNOS) resulting in NO-mediated nitrosative stress [7]. Interestingly, NO has been shown to impede chondrocyte survival and induces cell death [1]. All in all, these reports highlight the important of FD in the acquisition of apoptosis in chondrocytes. However, information pertaining to the effects of FD on the functional attributes of synoviocytes and its possible involvement in the pathogenesis of OA is spares. For this reason, this study will focus on the aspect of FD in the survival of synoviocytes, and the role of ROS and to identify the originating sites of mitochondrial respiratory chain (MRC) using various site specific inhibitors targeting various complexes localized in MRC. Unrevealing the underlying mechanism(s) of cell demise by FD are also within the scope of this investigation.

## Materials and Methods

### Cell line and reagents

HIG-82 cell line (rabbit synoviocytes) and HeLa cell line were obtained from the Bioresource Collection and Research Center (Hsinchu, Taiwan). The folate deficient medium powder used in this study was purchased from GIBCO for which folate as well as thymidine, hypoxanthine, and glycine were omitted from complete media to stress substrate availability in one carbon metabolism. To minimize exogenous folate source, fetal bovine serum was replaced with dialyzed fetal bovine serum (dFBS). Control medium was complete medium with 10% FBS. Dichlorofluorescein diacetate (DCFH-DA) and chloromethylflourescein diacetate (CMF-DA) were acquired from Invitrogen Co. (Carlsbad, CA). FITC-IETD-FMK and FITC-LEHD-FMK were obtained from United States Biological (Swampscott, MA). The primary antibodies against caspase 3, gp91, p22 and second antibodies were obtained from Santa Cruz Biotechnology, Inc., (Santa Cruz, CA). The primary antibodies against β-actin, JC-1, Fluo 3-AM and other chemicals were purchased from Sigma Chemical Co. (St. Louis, MO).

### Cell culture and treatment

HIG-82 synoviocytes (1×10^6^) were cultured in an F-12 medium supplemented with 10% fetal bovine serum, 100 U/ml penicillin and 100 lg/ml streptomycin in 100-mm cultured dishes at 37°C in a humidified atmosphere of 5% CO_2_. HeLa cells (1×10^6^) were cultured in a DMEM/F-12 1:1 medium supplemented with 10% fetal bovine serum, 100 U/ml penicillin and 100 lg/ml streptomycin in 100-mm cultured dishes at 37°C in a humidified atmosphere of 5% CO_2_. When cells reach 80% confluence in 100-mm cultured dishes, the cells were washed with PBS and trypsinized to use in various experiments. The selective inhibitors and its particular concentrations used to inhibit the intracellular ROS generation from various enzymes were referenced and modified from our previous publication [25]. These particular concentrations of selective inhibitors did not affect the cell viability of HIG-82 cells. The selective inhibitor of mitochondrial complex I (rotenone 20 nM), mitochondrial complex II (carboxin 5 μM or TTFA 5 μM), mitochondrial complex III (antimycin A 0.01 nM) and NADPH oxidase (AEBSF 2 μM or apopcynin 30 μM) were pretreated 2 h, followed by FD treatment for 48 h. Calcium chelator (BAPTA 5 μM) was pretreated 3 h, followed by FD treatment for 48 h. These inhibitors were purchased form Sigma Chemical Co. (St. Louis, MO).

### Cell viability assay

Cell viability was assessed by the MTT assay. The MTT (Sigma-Aldrich, St. Louis, MO, USA) is reduced to purple formazan by dehydrogenase in the mitochondria of living cells. HIG-82 synoviocytes (3×10^4^) were plated in 12-well cultured plates for 24 h. The culture medium was replaced with three types of media: (1) Control F12 culture medium containing folate supplemented with 10% undialyzed fetal bovine serum (FBS), designated as FC medium. (2) Marginal folate deficient medium containing folate, but supplemented with dialyzed FBS (dFBS), designated as MFD medium. (3) Folated deficient medium containing no folate, thymidine, hypoxanthine, glycine and supplemented with 10% of dFBS, designated as FD medium. Other experimental groups were MFD plus 1 μM folate and FD plus 1 μM folate. Synoviocytes cultivated with these types of media were allowed to grow for 1, 2 and 3 days. The viability of the cells was then determined by MTT test. Cells were incubated with 500 μl of MTT solution (0.5 mg/ml) for 2 hr at 37°C, and the solution was replaced with 500 μl DMSO after the incubation. The absorbance of DMSO lysed solution was measured at OD 575 nm [26]. The data were acquired, analyzed and plotted by the Sigma Plot 10.0 software.

### Measurement of intracellular ROS by flow cytometry

Production of intracellular ROS was detected by flow cytometry using DCFH-DA probe (Sigma-Aldrich, St. Louis, MO, USA). HIG-82 synoviocytes (1.5×10^5^) were plated in 6-cm culture dishes for 24 h. The culture medium was replaced with three types of media: (1) FC medium, (2) MFD medium and (3) FD medium for 48 h. Cells were treated with 10 μM DCFH-DA for 30 min in the dark, washed once with PBS, collected by centrifugation, and then suspended in PBS. Intracellular ROS levels indicated by the fluorescence of dichlorofluorescein (DCF) were evaluated by excitation at 488 nm and measured through a 530/22-nm barrier filter using a Becton-Dickinson FACSan flow cytometer [25]. The data were acquired, analyzed and plotted by the CellQuest Pro software and the Sigma Plot 10.0 software.

### Measurement of intracellular calcium levels by flow cytometry

Intracellular calcium levels were detected by flow cytometry using Fluo3-AM probe (Invitrogen, CA, USA). HIG-82 synoviocytes (1.5×10^5^) were plated in 6-cm culture dishes for 24 h. The culture medium was replaced with three types of media: (1) FC medium, (2) MFD medium and (3) FD medium for 48 h. After treatment, cells were trypsinized by trypsin, treated with 2 μM Fluo3-AM for 30 min in the dark, washed twice with PBS, collected by centrifugation, and then suspended in PBS. Intracellular calcium levels were evaluated by excitation at 488 nm and measured through a 530/22-nm barrier filter using a Becton-Dickinson FACSan flow cytometer [27]. The data were acquired, analyzed and plotted by the CellQuest Pro software and the Sigma Plot 10.0 software.

### Measurement of intracellular GSH depletion

HIG-82 synoviocytes (1.5×10^5^) were plated in 6-cm culture dishes for 24 h. The culture medium was replaced with three types of media: (1) FC medium, (2) MFD medium and (3) FD medium for 48 h. After treatment, cells were incubated with 5 μM CMF-DA for 20 min at 37°C in a 5% CO_2_ incubator, washed once with PBS, collected by centrifugation, suspended in PBS, and then measured through a 530/22-nm barrier filter using a Becton-Dickinson FACSan flow cytometer. The CMF fluorescence gives a measure of the intracellular GSH level. The low CMF fluorescence represents the cellular percentages of GSH depletion [26]. The data were acquired, analyzed and plotted by the CellQuest Pro software and the Sigma Plot 10.0 software.

### Apoptosis and cell cycle analysis

Apoptosis and cell cycle were measured with propidium iodide (PI) staining and flow cytometry. HIG-82 synoviocytes (1.5×10^5^) were plated in 6-cm culture dishes for 24 h. The culture medium was replaced with three types of media: (1) FC medium, (2) MFD medium and (3) FD medium for 48 h. After treatment, cells were collected, washed with PBS, fixed in PBS-methanol (1:2, volume/volume) solution, and then maintained at 4°C for at least 18 h. After once wash with PBS, the cell pellets were stained with a PI solution containing PBS, PI (40 μg/mL) and DNase-free RNase A (40 μg/mL) for 30 min at room temperature in the dark. The cell pellets were then analyzed using a Becton-Dickinson FACSan flow cytometer (Franklin Lakes, NJ). PI is an ***intercalating agent and a fluorescent molecule that stains double-stranded DNA. In methanol-fixed cells, the PI molecules translocate into the nucleus and bind to the double-stranded DNA. The PI fluorescent intensity in apoptosis cells was weaker than that of cells in the G1 phase. The percentage of apoptosis cells was characterized as the percentage of cells in the SubG1 region of the DNA distribution histograms. A minimum of 1 × 10^4^ cells was counted per sample [25]. The data were acquired, analyzed and plotted by the CellQuest Pro software and the Sigma Plot 10.0 software.

### Measurement of mitochondrial membrane permeability

The mitochondrial membrane permeability transition event in whole cell samples was used the JC-1 potentiometric dye. HIG-82 synoviocytes (1.5×10^5^) were plated in 6-cm culture dishes for 24 h. The culture medium was replaced with three types of media: (1) FC medium, (2) MFD medium and (3) FD medium for 48 h. After treatment, the cells were trypsinized, and then incubated with 15μM JC-1 for 10 min at 37°C in a CO_2_ incubator. This cyanine dye accumulates in the mitochondrial matrix under the influence of the mitochondrial membrane potentials and forms JC-1 aggregates, which have characteristic absorption and emission spectra. Once membrane potentials decrease, JC-1 aggregates depart from mitochondrial matrix and change to JC-1 monomers, in the meantime, JC-1 changes color from orange to green. Reversible formation of JC-1 aggregates causes a shift of emitted light from 530 nm to 590 nm. Following the incubation step, the changed fluorescence level of JC-1 was analyzed using a Becton-Dickinson FACScan flow cytometer [28]. The data were acquired, analyzed and plotted by the CellQuest Pro software and the Sigma Plot 10.0 software.

### TUNEL assay

HIG-82 synoviocytes (1.5×10^5^) were plated in 6-cm culture dishes for 24 h. The culture medium was replaced with three types of media: (1) FC medium, (2) MFD medium and (3) FD medium for 48 h. Cells were fixed in 1% paraformaldehyde in PBS for 30 min, then washed with PBS, and stored in 70% methanol at 4°C. After rehydration in PBS, cells were assayed with Apoptosis Detection Kit (APO-BRDU) (BD Pharmigen) [29]. The data were acquired, analyzed and plotted by the CellQuest Pro software and the Sigma Plot 10.0 software.

### Western blotting analysis

HIG-82 synoviocytes (1.5×10^5^) were plated in 6-cm culture dishes for 24 h. The culture medium was replaced with three types of media: (1) FC medium, (2) MFD medium and (3) FD medium for 48 h. After treatment, cells were washed with PBS, resuspended in a protein extraction buffer for 10 min, and centrifuged at 12,000g for 10 min at 4°C to obtain total extracted proteins (supernatant). Protein concentrations were measured with a Bio-Rad protein assay reagent (Bio-Rad, Richmond, CA). The extracted cellular proteins were boiled in loading buffer, and an aliquot corresponding to 60 μg of protein was separated on a 12% SDS-polyacrylamide gel. After electrophoresis, proteins were electrotransferred onto a polyvinylidene fluoride transfer membrane. After blotting, the membranes were incubated with various primary antibodies overnight and then washed with PBST solution (0.05% Tween 20 in PBS). Following washing, the secondary antibody labeled with horseradish-peroxidase was added to the membrane for 1 h and then washed with PBST solution (0.05% Tween 20 in PBS). The antigen-antibody complexes were detected by enhanced chemiluminescence (Amersham Pharmacia Biotech, Piscataway, NJ) with a chemiluminescence analyzer [25].

### HPLC quantitative analysis of folic acid in cells and media

HIG-82 synoviocytes (3×10^5^) were plated in 100-mm cultured dishes for 24 h. The culture medium was replaced with FC medium, MFD medium or FD medium. Synoviocytes cultivated with these three types of media were allowed to grow for 48 h. After treatment, the cells were collected, twice washed with PBS, added 1 mL MeOH/PBS (7/3, v/v) using ultrasonic cell disruption for 30 min, then was filtered through a 0.47 μm filter. The cells lysed samples were used to measured folate concentrations using HPLC followed by UV (280nm) detection. The concentrations of folate in media and cells were evaluated by HPLC, respectively. For HPLC analysis using with RP-C_18_ column (4.6 mm × 250 mm, 5 μm, Merck, Germany), a mobile phase consisting of 40 mM sodium phosphate dibasic heptahydrate buffer, and 5% acetonitrile (v/v), pH 5.5. The mobile phase was filtered through a 0.47 μm filter and then deaerated ultrasonically prior to use. Folate was quantified by a UV detector at the wavelength of 280 nm following HPLC separation. Flow rate was 1.0 mL/min, the injection volume was 10 μL and the column temperature was maintained at 25°C. The chromatographic peak of the analyte was confirmed by comparing its retention time (tR 15.8 ± 0.2 min) with the reference standard. Quantification was carried out by the integration on area under curve (AUC) of the peak using external standard method. The working calibration curve based on folic acid standard solutions showed good linearity over the range of 0.078–10 μg/mL. The regression line for folate was y = 34599x − 1019.6 (R2 = 0.9999), where y is the peak area of folate, and x is the concentration (μM) [30]. The data were acquired, analyzed and plotted by the Sigma Plot 10.0 software.

### Measurement of caspase 8 and caspase 9 activities by flow cytometry

The caspase substrates, FITC-IETD-FMK for caspase 8 and FITC-LEHD-FMK for caspase 9, were diluted with a buffer to make the desired concentrations of various homogeneous substrate reagents. After treatment, the cells were washed once with PBS, detached by trypsinization, and collected by centrifugation. Aliquot 1×10^5^ cells were suspended in an F-12 medium, and then various homogeneous substrate reagents were added to the cells, maintaining a 1:1 ratio of reagent to cell solution. After 1 h of incubation at 37°C, the cells were washed once with PBS, collected by centrifugation, and suspended in PBS. FITC-IETD-FMK and FITC-LEHD-FMK are cell permeable, nontoxic, and irreversibly which can bind to activated caspase 8 and caspase 9 in apoptotic cells, respectively. The FITC label allows detection of activated caspase 8 and caspase 9 in apoptotic cells directly by flow cytometry with excitation wavelength set at 488 nm and emission wavelength at 520 nm [25]. The data were acquired, analyzed and plotted by the CellQuest Pro software and Sigma Plot 10.0 software.

### Statistic analysis

Data are presented as the mean (SD) of at least 3 independent experiments and were analyzed using Student’s *t*-test by the Sigma Plot 10.0 software. A *P* value < 0.05 was considered statistically significant.

## Results

### FD impedes the growth of synoviocytes

**Fig 1:**
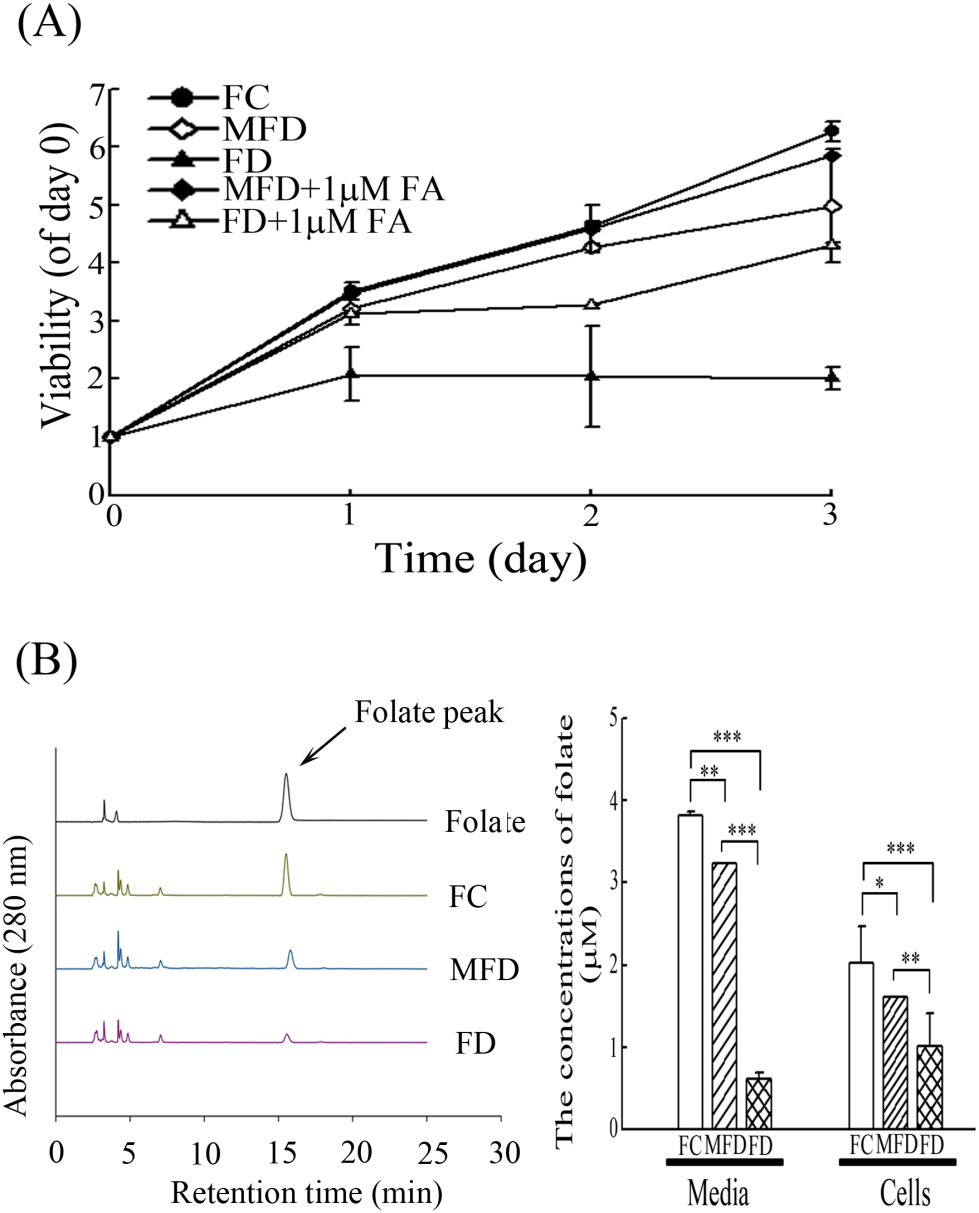
Folate deficiency impedes the growth of synoviocytes. (A) HIG-82 synoviocytes (3×10^4^) or (B) HIG-82 synoviocytes (3×10^5^) were plated in 12-well culture plates or 100-mm cultured dishes, respectively, for 24 h. The culture medium was replaced with three types of media: (1) Control F12 culture medium containing folate supplemented with 10% undialyzed fetal bovine serum (FBS), designated as FC medium. (2) Marginal folate deficient medium containing folate, but supplemented with dialyzed FBS (dFBS), designated as MFD medium. (3) Folated deficient medium containing no folate, thymidine, hypoxanthine, glycine and supplemented with 10% of dFBS, designated as FD medium. Other experimental groups were MFD plus 1 μM folate (FA) and FD plus 1 μM folate. (A) Synoviocytes cultivated with these types of media were allowed to grow for 1, 2 and 3 days. The viability of the cells was then determined by MTT assay. (B) Synoviocytes cultivated with FC, MFD, and FD media and allowed to grow for 48 h. The concentrations of folate in media and cells were evaluated by HPLC. Illustrate chromatograms of the folate studied the analyzed cell lysed samples (FC, FD, and MFD medium). The retention time for folate was tR 15.8 ± 0.2 min. LC C-18 column 5 μm (250 mm × 4.6 mm) and a mobile phase, consisting of 40 mM sodium phosphate dibasic, heptahydrate buffer, and 5% acetonitrile (v/v), pH 5.5. The values shown are expressed as mean ± SD (n = 5–8 samples per experiment). Significant differences from the FC group are *p*<0.05 (*), *p*<0.01 (**), *p*<0.001 (***).

When synoviocytes were cultivated in FC and MFD medium, a progressive time-dependent increment of cell growth for up to 72-h could be observed. In contrast, HIG-82 synoviocytes cultivated under FD condition, the growth rate of synoviocytes were found to be severely retarded (Fig 1A). The cell viability is partially recovered in MFD and FD by 1 μM of folate supplement in media. An experimental measure of folate (in cells and media) was incorporated. To suggest experimental thoroughness, support the accurate definition of the various culture conditions (FC, MFD vs FD) used in the study and ensure that the experimental regimen is indeed successful to achieve endogenous folate depletion. An experimental measure of folate (in cells and media) was incorporated. As shown in Fig 1B, the folate concentration in FD was decreased significantly in HIG-82 synoviocytes and media as compared with FC and MFD (S1 Table). These results ensure that the experimental regimen is indeed successful to achieve endogenous folate depletion.

### FD triggers apoptosis of synoviocytes

**Fig 2:**
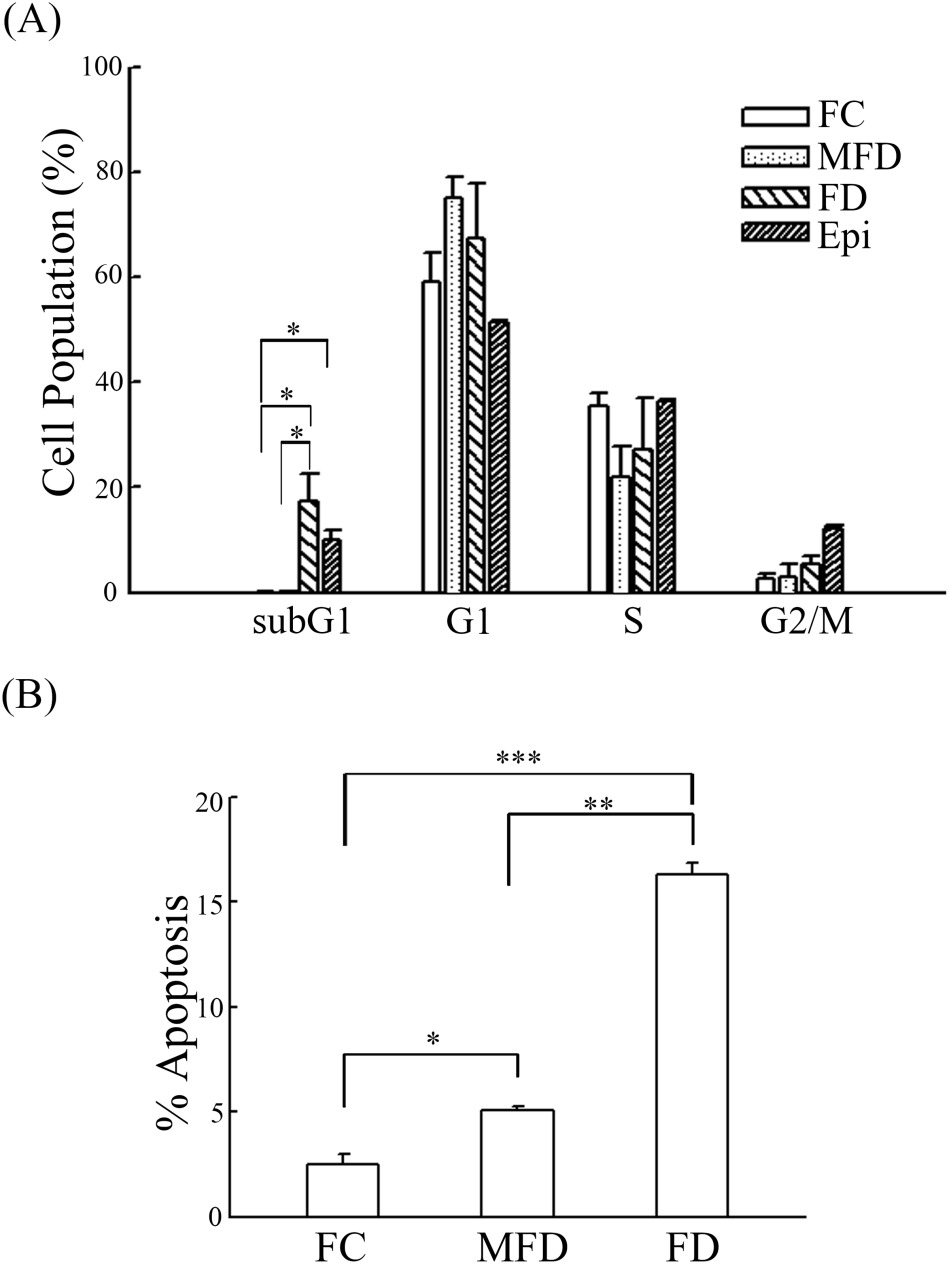
Folate deficiency provokes apoptotic lethality in synoviocytes. HIG-82 synoviocytes (1.5×10^5^) were plated in 60-mm cultured dishes for 24 h. The culture medium was replaced with FC, MFD, and FD media and then continued cultivating for additional 48 h. (A) Cells were then collected, washed with PBS, fixed in PBS-methanol (1:2 v/v) solution and maintained at 4°C for at least 18 h. After one washed with PBS, the cell pellets were then stained with a PI solution containing PBS, PI (40μg/mL), and DNase-free RNase A (40μg/mL) for 30 min at RT in the dark. The cell pellets were then analyzed using a Becton-Dickinson FACSan flowcytometer. The epirubicin (500 nM) treatment (Epi) is a positive control assay of apoptosis. The blank bar, gray bar, right slash bar and left slash bar represent FC, MFD, FD and Epi treatment, respectively. The percentages of subG1 population determined by the PI fluorescent intensity in apoptosis cells which was weaker than that of cells in the G1 phase. The percentages of apoptosis cells were characterized as the percentages of cells in the SubG1 region of the DNA distribution histograms. The FD subG1 bar graph is compared with FC or MFD. A *p*<0.05 (*) was considered statistically significant. (B) Cells were fixed in 1% paraformaldehyde in PBS for 30 min, then washed with PBS, and stored in 70% methanol at 4°C. After rehydration in PBS, cells were evaluated with TUNEL assay. The values shown are mean ± SD (n = 5–8 samples per experiment). Significant differences from the FC or MFD groups are *p*<0.05 (*), *p*<0.01 (**), *p*<0.001 (***).

Two different types of experiments were conducted in order to ascertain that FD-induced cell demise was apoptotic in nature. First, we performed cell cycle analysis and found that the percentage of sub G1 fraction (apoptosis) of HIG-82 synoviocytes grown in FD medium for 2-day rose significantly to 19.04±4.3%. Comparatively, the sub G1 fractions of synoviocytes cultivated under either MFD or FC condition were relatively minimal (0.13±0.09% and 0.10±0.05%, respectively) (Fig 2A and S1 Fig). Second, we also performed TUNEL assay and found that TUNEL-positive fraction of HIG-82 synoviocytes cultivated under FD condition was significantly higher than those either grown under MFD or FC controls (15.43±0.53% vs 4.17±0.58% and 3.90±0.77%, respectively) (Fig 2B and S2 Fig).

### FD provokes increased ROS production and triggers elevated calcium release

**Fig 3:**
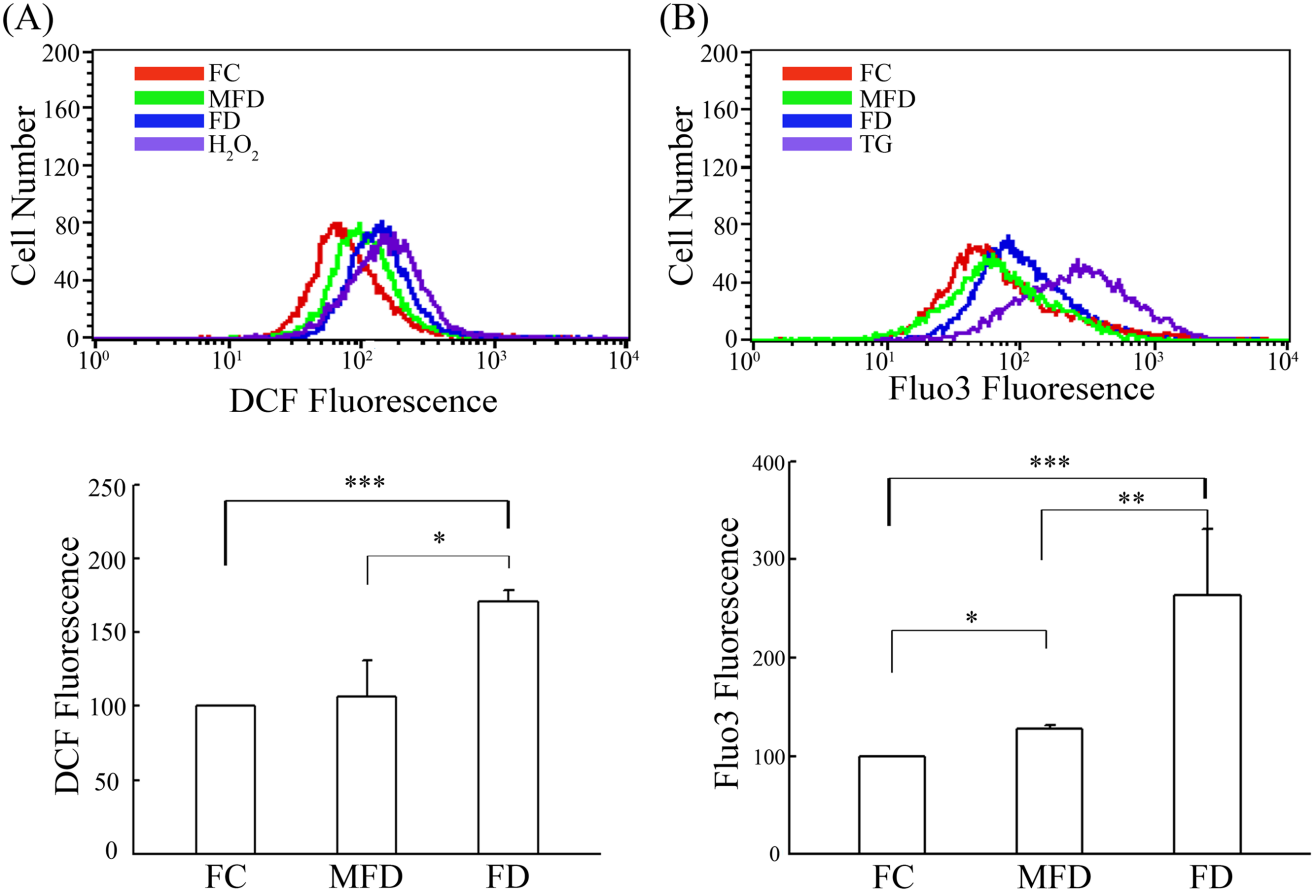
Folate deficiency triggers increased production of ROS and plethorically release of intracellular calcium levels. HIG-82 synoviocytes (1.5×10^5^) were plated in 60-mm cultured dishes for 24 h. The cultured media were replaced with FC, MFD, and FD media and then continued cultivating for additional 48 h. H_2_O_2_ (400μM) treatment for 30 min and thapsigargin (TG; 10μM) treatment for 10 min were the positive control groups of ROS and intracellular calcium, respectively. (A) Intercellular ROS was detected flowcytometrically using DCFH-DA staining. The red line, green line, blue line and purple line represent FC, MFD, FD and H_2_O_2_ treatments, respectively. (B) Intracellular calcium concentration was also measured flowcytometrically using Fluo-3 AM staining. The red line, green line, blue line and purple line represent FC, MFD, FD and TG treatments, respectively. The peaks in each panel represent the mean fluorescence intensities. The values shown are mean ± SD (n = 5–8 samples per experiment). Significant differences from the FC or MFD groups are *p*<0.05 (*), *p*<0.01 (**), *p*<0.001 (***).

ROS production of synoviocytes cultivated under FC, MFD or FD condition was evaluated flowcytometrically using DCFH-DA as the probe. As compared to the FC control, ROS generated by FD group was nearly two-fold higher than FC control as reflected by the DCF fluorescent intensity being measured (Fig 3A). In parallel, intracellular calcium levels were also evaluated flowcytometrically using fluo3-AM as the probe. Again, as compared to the FC control, there was a nearly 3-fold increase of intracellular calcium levels being detected in FD group (Fig 3B). The histograms are rather broad indicating that the cellular calcium might be measured in two different populations of cells. These data implicate that FD-induced ROS production serves as a mediator for intracellular calcium release.

### Evidence that FD-evoked ROS production is originated from mitochondrial complex II and NADPH oxidase

**Fig 4:**
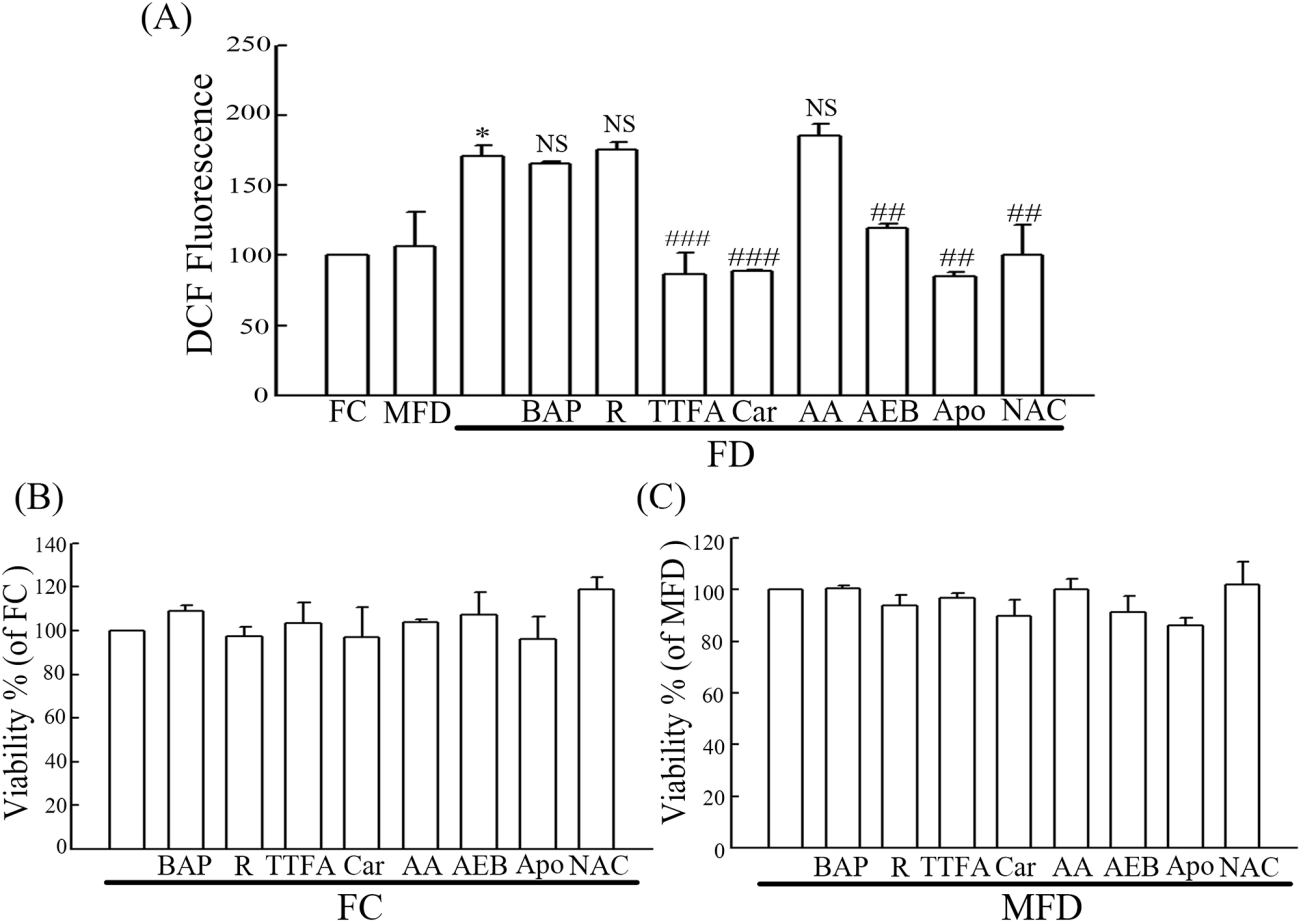
Identification of originating sites of FD-evoked ROS overproduction using a group of specific inhibitors. HIG-82 synoviocytes (1.5×10^5^) were plated in 60-mm cultured dishes for 24 h. (A) HIG-82 synoviocytes were separately grown under FC, MFD and FD media. Except for FC and MFD groups, cells in FD group were pretreated with or without various concentrations of designated inhibitors including rotenone (R, mitochondrial complex I; 20 nM), 2-thenoyltrifluoroacetone (TTFA, mitochondrial complex II; 5 μM), carboxin (Car, mitochondrial complex II; 5μM), antimycin A (AA, mitochondrial complex III; 0.01 nM), 4-(2-Aminoethyl)benzenesulfonyl fluoride hydrochloride (AEB, NADPH oxidase; 1μM), apocynin (Apo, NADPH oxidase; 30μM), and *N*-acetylcysteine (NAC, antioxidant; 20 mM) for 2 h. BAPTA (BAP, Ca^2+^ chelator; 5μM) was used as the control and pretreatment duration was 3 h. Intracellular ROS production was then detected flowcytometrically using DCFH-DA staining. (B) and (C) The inhibitor treatments in FC and MFD growth conditions were evaluated by MTT assay. The values shown are mean ± SD (n = 5–8 samples per experiment). Significant differences from the FC group are *p*<0.05 (*) and the untreated FD group are *p*<0.01 (##), *p*<0.001 (###), respectively. The NS represents no significant difference from the untreated FD group.

To identify the possible originating sites of elevated ROS generation instigated by FD condition, we employed various ROS inhibitors. As indicated in Fig 4A, we demonstrated that FD-evoked ROS production could not be suppressed by inhibitors of mitochondrial complex I (rotenone) and mitochondrial complex III (antimycin A). Conversely, we found that inhibitors specific for mitochondrial complex II (TTFA and carboxin) and NADPH oxidase (AEBSF and apocynin) could effectively suppress FD-instigated ROS overproduction. Interestingly, BAPTA, a Ca^2+^ chelator, was found to be incapable of inhibiting FD-instigated ROS production indicating that intracellular Ca^2+^ release may be a downstream event of ROS generation. In addition, we provided comparative data for inhibitor treatments in FC and MFD growth conditions. In Fig 4B and 4C, the cell viabilities in all inhibitor treatments in FC and MFD were maintained above 85%. Taken together, our data clearly indicate that FD-evoked ROS overproduction is originated from NADPH oxidase and mitochondrial complex II.

### Inhibition of FD-evoked ROS production concomitantly suppresses intracellular calcium release and attenuates apoptosis of synoviocytes

**Fig 5:**
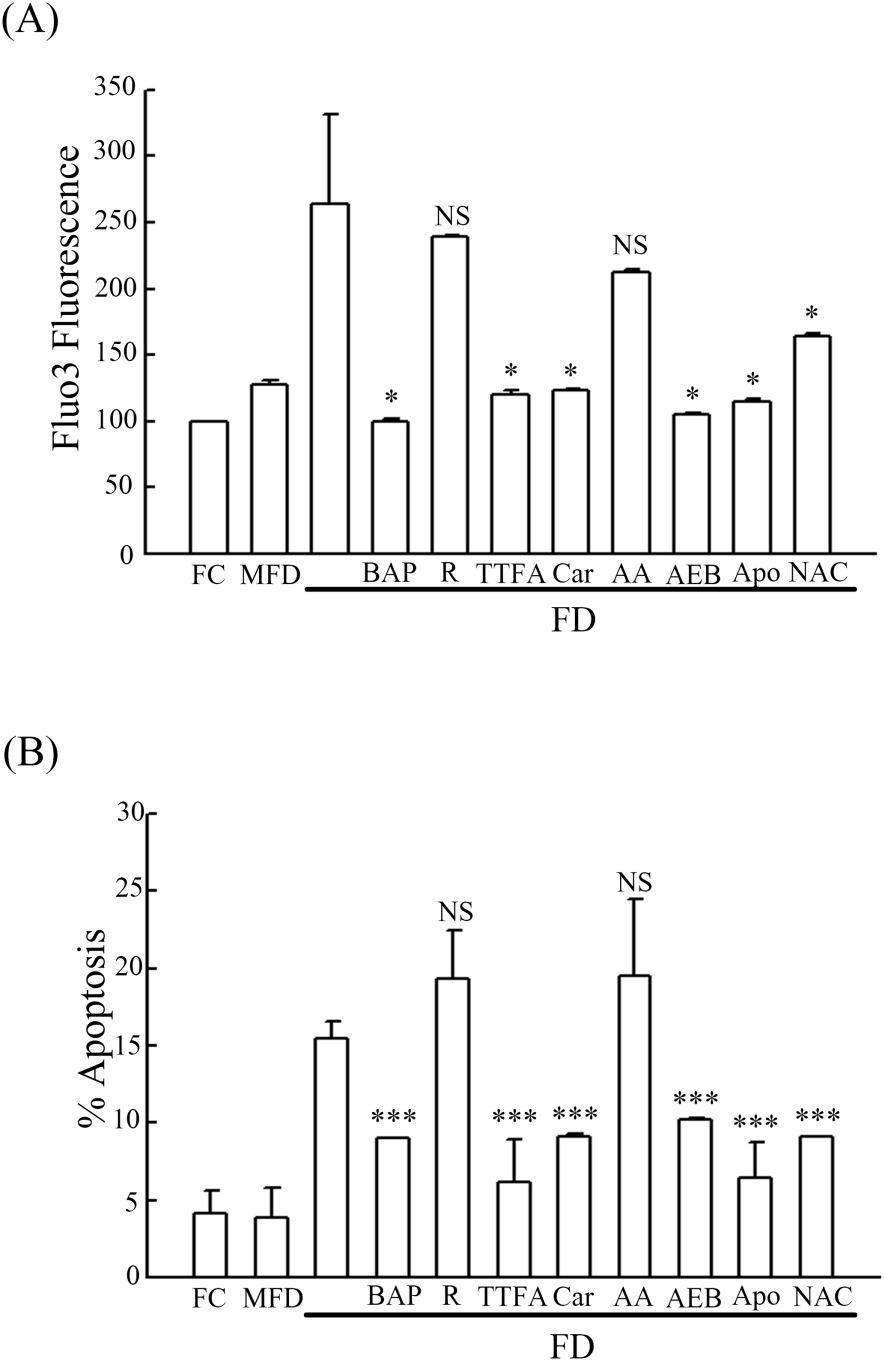
Effects of site-specific inhibitors on intracellular Ca^2+^ release and their resultant consequences on the extents of apoptotic lethality in FD-cultivated synoviocytes. (A) HIG-82 synoviocytes were handled as those described in Fig 4. (A) The Ca^2+^ concentration being released was measured flowcytometrically using Fluo-3 AM staining. (B) The corresponding apoptosis indices in (A) were determined using TUNEL assay. Significant differences from the untreated FD group are *p*<0.05 (*) and *p*<0.001 (***). The NS represents no significant difference from the untreated FD group.

**Fig 6:**
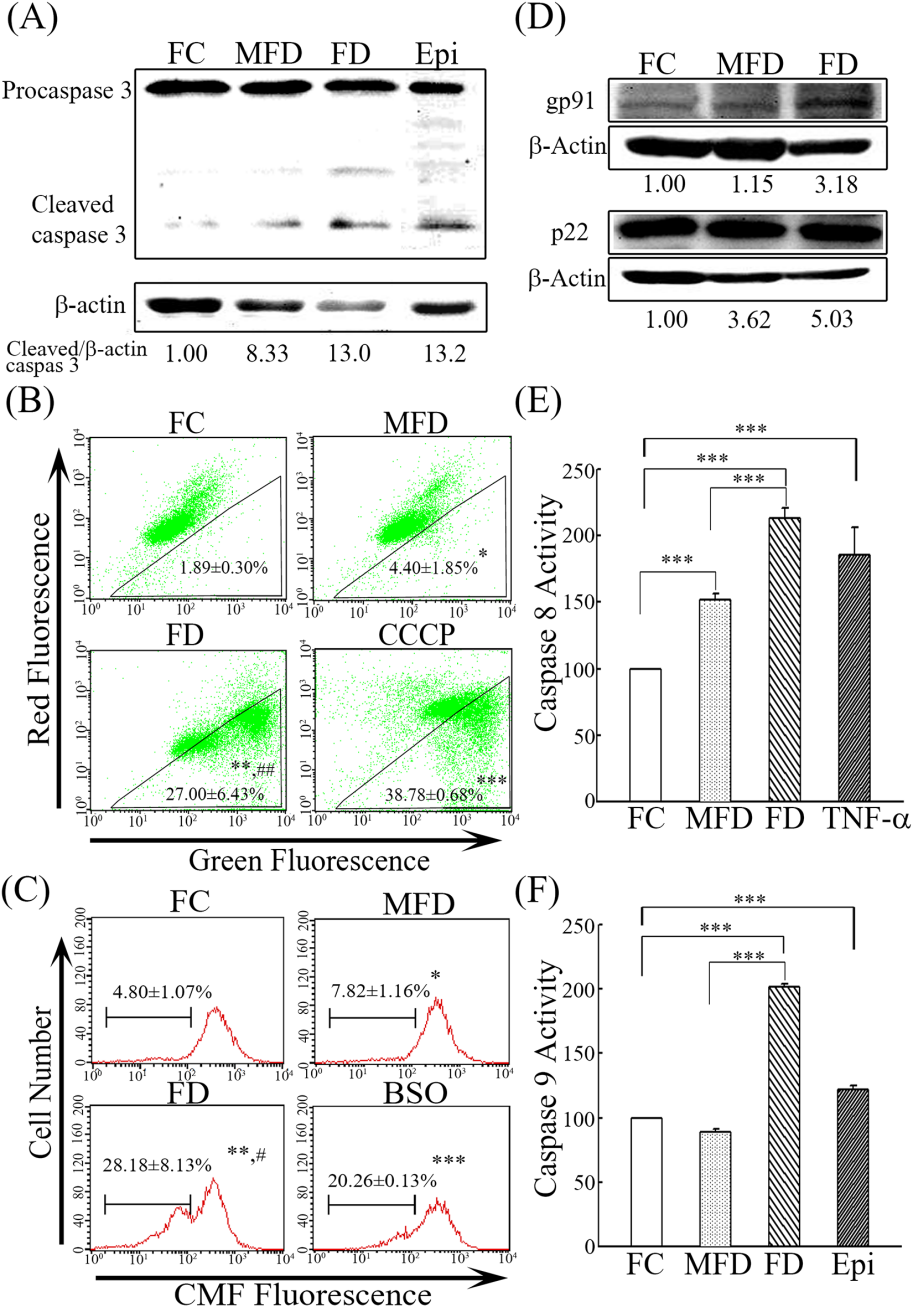
The alternate biochemical experiments to test for apoptosis under all the growth conditions and markers for oxidative stress. (A) Caspase 3 activation after treatment with FC, MFD, FD and epirubicin (Epi). HIG-82 synoviocytes (3×10^5^/100-mm cultured dishes) were treated with FC, MFD and FD for 48 h or 500 nM Epi (a positive control) for 48 h. The cleaved caspase 3 represented the caspase 3 activation was determined by western blotting. (B) Mitochondrial transmembrane potential disruption after treatment with FC, MFD, FD and CCCP. HIG-82 synoviocytes (1.5×10^5^/60-mm cultured dishes) were treated with FC, MFD and FD for 48 h or 200μM CCCP (a positive control) for 24 h. After treatment, the culture medium was replaced with a new medium with 15 μM JC-1 for 20 min in the dark. Bivariate plots of red versus green fluorescence shows an evaluation of mitochondrial transmembrane potential. Values in each box express cellular percentages of decreased mitochondrial transmembrane potential. The values shown are mean ± standard deviation (n = 5–8). Significant differences from the FC group are *p*<0.05 (*), *p*<0.01 (**), *p*<0.001 (***) and the MFD group are *p*<0.01 (##). (C) Glutathione depletion after treatment with FC, MFD, FD and buthionine sulphoximine (BSO). HIG-82 synoviocytes (1.5×10^5^/60-mm cultured dishes) were treated with FC, MFD, FD or 0.5 mM BSO (a positive control) for 48 h. The cellular percentages of glutathione depletion were evaluated by CMF-DA staining and flow cytometry. Data show the percentages of cells displaying intracellular GSH depletion. The values shown are mean ± standard deviation (n = 5–8). Significant differences from the FC group are *p*<0.05 (*), *p*<0.01 (**), *p*<0.001 (***) and the MFD group are *p*<0.05 (#). (D) Expressions of gp91 and p22 after treatment with FC, MFD, FD. HIG-82 synoviocytes (3×10^5^/100-mm cultured dishes) were treated with FC, MFD and FD for 48 h. The expressions of gp91 and p22 were determined by western blotting. (E) Caspase 8 activation after treatment with FC, MFD, FD and TNF-α. HIG-82 synoviocytes (1.5×10^5^/60-mm cultured dishes) were treated with FC, MFD and FD for 48 h or 100 ng/ml TNF-α (a positive control) for 24 h. The caspase 8 activation was determined as outlined in Materials and Methods. The values shown are mean ± standard deviation (n = 5–8). Significant differences are P<0.001 (***). (F) Caspase 9 activation after treatment with FC, MFD, FD and epirubicin (Epi). HIG-82 synoviocytes (1.5×10^5^/60-mm cultured dishes) were treated with FC, MFD and FD for 48 h or 500 nM Epi (a positive control) for 24 h. The caspase 9 activation was determined as outlined in Materials and Methods. The values shown are mean ± standard deviation (n = 5–8). Significant differences are P<0.001 (***).

In this study, we first demonstrated that FD-evoked ROS production in synoviocytes could not be suppressed by inhibitors specific for mitochondrial complex I (rotenone) and complex III (antimycin A). Concomitantly, both inhibitors were found to be incapable of curtailing the release of intracellular calcium levels and attenuating the magnitude of apoptosis. In contrast, we uncovered that FD-evoked overproduction of ROS could be strongly suppressed by inhibitors of NADPH oxidase (AEBSF and apocynin) and Complex II (TTFA and carboxin). This phenomenon was accompanied with the drastic reduction of intracellular calcium release (Fig 5A). Consequently, FD-induced apoptotic lethality could be effectively attenuated (Fig 5B). Our data clearly confirm that FD-evoked ROS production can serve as a mediator for the production of intracellular calcium release that eventually fostering the occurrence of apoptosis of synoviocytes. Finally, the alternate biochemical experiments to test for apoptosis under all the growth conditions and markers for oxidative stress were evaluated. In Fig 6A and 6E, the caspase 3 (an executioner caspase) and caspase 8 (an initiator caspase) were activated in MFD and FD treatments as compared with FC treatment (S3 Fig). The caspase 9, an initiator caspase, was activated in FD treatment but not in MFD treatment (Fig 6F and S4 Fig). The mitochondrial transmember potential disruption was significantly increased to 27% of cells in FD treatment as compared with the FC and MFD treatments which were less than 7% (Fig 6B). Glutathione depletion and expressions of gp91 and p22 (two NADPH oxidase subunits) are two markers of oxidative stress [31,32]. In FC and MFD treatments, the GSH depletion was less than 10% of cells (Fig 6C). FD treatment resulted in 28% of GSH depletion (Fig 6C). In Fig 6D, the expressions of gp91 and p22 was increased to about 3.2-fold and 5.0-fold in FD treatment, respectively, as compared with FC treatment.

### Effect of FD on the growth of HeLa cells

**Fig 7:**
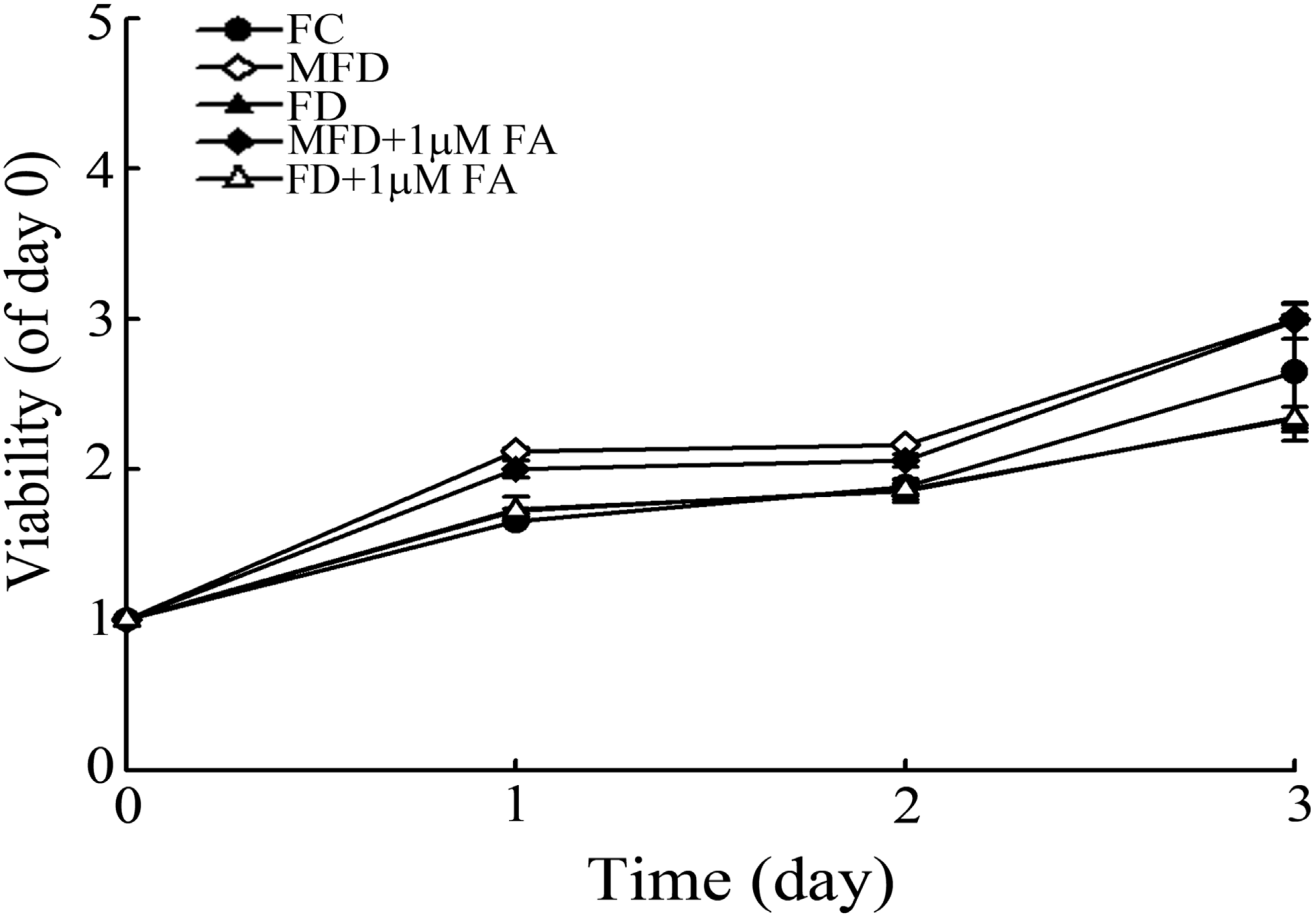
Folate deficiency does not impede the cell growth in HeLa cells. HeLa cells (3×10^4^) were plated in 12-well culture plates for 24 h. The culture medium was replaced with three types of media: (1) Control DMEM/F-12 1:1 medium containing folate supplemented with 10% undialyzed fetal bovine serum (FBS), designated as FC medium. (2) Marginal folate deficient medium containing folate, but supplemented with dialyzed FBS (dFBS), designated as MFD medium. (3) Folated deficient medium containing no folate, thymidine, hypoxanthine, glycine and supplemented with 10% of dFBS, designated as FD medium. Other experimental groups were MFD plus 1 μM folate (FA) and FD plus 1 μM folate. (A) Synoviocytes cultivated with these types of media were allowed to grow for 1, 2 and 3 days. The viability of the cells was then determined by MTT assay. The values shown are expressed as mean ± SD (n = 5–8 samples per experiment).

To evaluate the effect of FD on regular laboratory HeLa cell line, the HeLa cells were cultivated in FC, MFD and FD media. As shown in Fig 7, the cell viability increases in a time-dependent manner in all experimental groups. It is indicating that FD does not impede the cell growth in HeLa cancer cells. Cancer cells may use some survival mechanisms to against FD.

## Discussion

Folate deprivation (FD) is prevalent in many kinds of disorders. Osteoarthritis (OA), mainly resulting from the regression of cartilage, chronic inflammation of the synovium and the subchondral bone remodeling. Recently, other studies demonstrate that cadherin-11 involves in synovitis and increases the migratory and invasive capacity of fibroblast-like synoviocytes of osteoarthritis [33]. Other reports also indicate that interleukin-1β up-regulates the expressions of intercellular adhesion molecule-1 and vascular cell adhesion molecule-1 in osteoarthritis fibroblast-like synoviocytes via nuclear factor -κB-mediated mechanism [34]. Enhancement of leukocytes infiltration and up-regulation of proinflammatory mediators play a crucial role in OA pathophysiology [34]. The effects of FD on synoviocytes *in vitro* remain unclear. Therefore, our study aimed to investigate whether FD resulted in effects on the HIG-82 synoviocytes.

The synoviocytes, a synovial intimal cell, are believed to be responsible for the production of synovial fluid components, for absorption from the joint cavity, and for blood/synovial fluid exchanges. Two types of synoviocytes, macrophagic cells (type A cells) and fibroblast-like cells (type B cells) have been identified. The type B cells, which are suitable synoviocytes, are involved in production of specialized matrix elements including hyaluronan, collagens and fibronectin for the intimal interstitium and synovial fluid. In some mammals, type B cells show characteristics suggesting sensory and endocrine functions, but these are not recognized in other species. The synoviocytes, which form a discontinuous cell layer, grow both fragmented basement membranes around the cells and junctional apparatus such as desmosomes and gap junctions [35]. HIG-82, a type B synoviocytes, is a continuous cell line isolated from soft tissue lining the knee joints of rabbits. This cell line was produced by spontaneous establishment of an aging, late-passage culture of primary cells. HIG-82 cells can be used as many experimental pathophysiologic models and activated by a number of stimuli, including phorbol myristate acetate, interleukin-1, and the endocytosis of latex beads. Activated HIG-82 cells secrete collagenase, gelatinase, caseinase, and prostaglandin E2 into their culture medium. The HIG-82 cell line should facilitate research into the biology and biochemistry of the fibroblastic cells. Such cells are likely to be important in the pathophysiology of some arthritis, including OA [36].

The folate metabolic pathway is important in several biological processes, including purine and pyrimidine synthesis and the methylation of DNA and proteins [37]. Reduced folate (tetrahydrofolate) is the proximal single carbon donor in several reactions involved in the de novo synthetic pathways for purine and pyrimidine precursors of DNA and RNA required for cell proliferation [38]. Furthermore, tetrahydrofolate plays a part in a second important biochemical step: the methionine homocysteine cycle, which is necessary to provide a methyl group for several downstream reactions such as methylation of DNA, RNA proteins, and others [38]. Once folate depletion in synoviocytes many cell functions will not normal process and results in cell death or inhibition of cell proliferation. Fig 1A shows an almost doubling of viability even in the FD grown cells. We speculate that there are little folate for cells to use from day 0 to day 1 which results in doubling of viability at day 1. However, the folate might complete deficiency resulted in the viability did not increase from day 1 to day 3. There are a large population is in G1 in the FD grown cells indicating that many cells were survival but stopped division.

Despite a plethora of literature has documented that prolific accumulation of ROS-mediated oxidative stress is believed to play a predominant role in the pathogenesis of OA as a result of an apoptotic lethality and matrix degradation of chondrocytes of articular cartilage [1,2,3,39], yet, information pertaining to possible involvement of synoviocyte functional abnormality in OA has been scanty. Along this same vein, since matrix degradation engendered in OA has been ascribable to the ROS-mediated activation of MMPs, one of the biochemical markers of epithelial-to-mesenchymal transition (EMT) that governing the migratory ability of an metastasis-prone cell types and was mediated by the activation of NF-κB transcription factor [40]. Coincidently, FD-instigated oxidative-nitrosative stress (ONS) observed in HepG2 cells is also associated with ROS triggered activation of NF-κB [24]. Based on the above-noted rationale, we thus hypothesize that FD episode could capacitate both chondrocytes and synoviocytes to activate MMPs through oxidative stress engendered by FD-triggered NF-κB activation. For this reason, we conducted the study here aiming to delineate whether synoviocytes cultivated under FD condition could poise themselves to apoptotic cell death. Furthermore, we also wanted to identify the originating site of ROS overproduction in MRC based on the previous report indicating that OA was associated with the mitochondrial dysfunction [1].

In our current study, we first demonstrated that synoviocyte (HIG-82) cell type cultivated under FD could induce cell growth impediment and triggered apoptotic lethality as evident by increased sub G1 fraction as well as elevated percentage of TUNEL-positive apoptotic cells. Further studies using site specific inhibitors of complexes in MRC, we were able to pinpoint the sites of the origin of ROS overproduction, namely: mitochondrial complex II and NADPH oxidase (NOX). Concomitantly, we also uncovered that ROS overproduction elicited cytosolic Ca^2+^ overload which was the downstream event of the former process. Both ROS overproduction and elevated Ca^2+^ released had been demonstrated to be the dual culprits for apoptotic lethality during an episode of FD condition. Interestingly, several literatures reported that folate deprivation-instigated diseases such as neural tube defects and congenital heart disease could be rescued by the supplementation strategy [13,41]. Under this premise, we speculate that folate supplementation strategy may be a preventive measure to rescue both chondrocytes and synoviocytes from FD-induced apoptosis and thus the risk of the occurrence of OA can be reduced.

Recently, Ralph et al. [42] reported that succinate dehydrogenase (SDH)/complex II system could act as a key redox regulator of ROS production via an electron driving mechanism. This study prompts us to investigate whether FD-evoked ROS overproduction can be similarly originated from SDH/complex II. This hypothesis turns out to be true since we utilize the site specific inhibitors of mitochondrial complex II (TTFA and carboxin) could strongly inhibit the ROS overproduction engendered by FD condition. In contrast, site specific inhibitors for complex I and III caused minimal effect on ROS production under similar FD condition. Along this same vein, we unexpectedly unveiled that ROS overproduction engendered by FD condition could also be strongly suppressed by NADPH oxidase (NOX) inhibitors (AEBSF and Apocynin). Our results are in accordance with the finding that folate supplementation could reduce homocysteine-induced superoxide anion (O_2_^−^) production via NADPH oxidase reported elsewhere [43,44]. Collectively, our data further strengthen the relevance of folate supplementation strategy as a preventive measure for the occurrence of OA.

It is worthy of noting that pretreatment of BAPTA, a chelator for Ca^2+^, could not inhibit ROS generation, but the pretreatment of synoviocytes with either complex II or NOX inhibitors could inhibit the elevation of cytosolic Ca^2+^. These data implicate that ROS generation is preceding to cytosolic Ca^2+^ release during an episode of FD condition. In line with our studies, Waypa et al [45] also demonstrated a significant increase in Ca^2+^ release during hypoxia, a situation mimicry to synoviocytes microenvironment could trigger mitochondrial ROS generation in pulmonary arterial myocytes. Ca^2+^ is a ubiquitous intracellular ion which acts as a signaling modulator in many cellular processes including cell proliferation, differentiation, survival and cell death [46]. FD-instigated cytosolic Ca^2+^ overload could thus be served as the arbitrator of apoptosis probably through the activation of Ca^2+^ dependent kinases and phosphatases [47].

**Fig 8:**
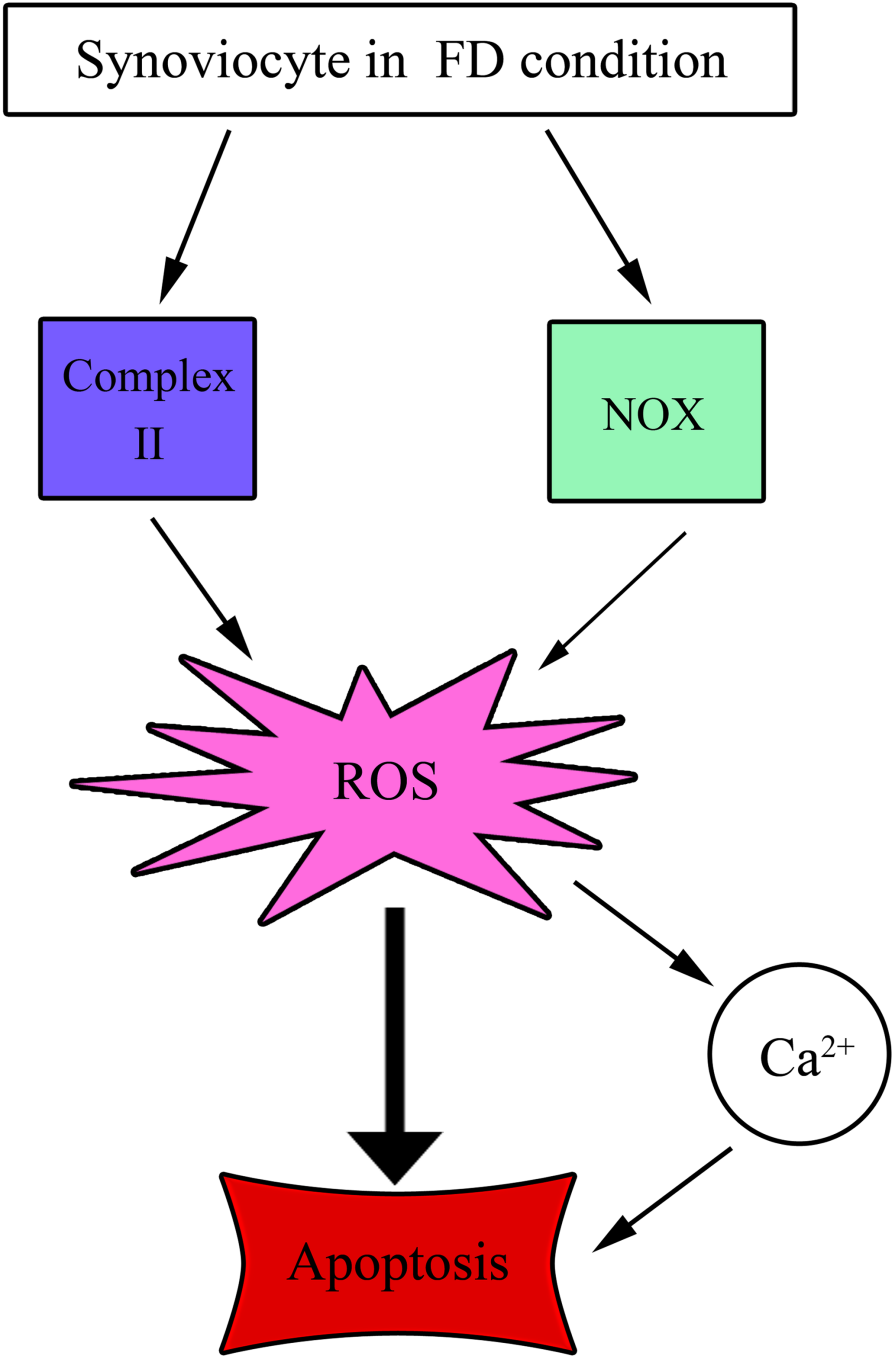
Diagrammatic scheme denoting the cascade of events that fostering folate deficient synoviocytes to apoptosis.

In conclusion, our current studies uncovered that synoviocytes cultivated under FD condition could elicit ROS overproduction and elevation of cytosolic Ca^2+^ release that triggered the occurrence of apoptotic lethality. Along the same vein, we first identified the site of MRC that initiated ROS overproduction being SDH/complex II, a major site for electron driving production of ROS during an episode of FD condition suggesting that inhibitors for complex II may be a targeting therapy to alleviate ROS production, Ca^2+^ overload and the extents of apoptosis. Lastly, we advocate the idea that folate supplementation strategy may be a suitable preventive measure for the occurrence of OA due to proper preservation of chondrocytes and synoviocytes without undergoing apoptotic lethality. Finally, a diagrammatic scheme depicting the cascade of events leading to FD-triggered apoptosis of synoviocytes can be seen in Fig 8.

## Supporting Information

S1 Fig Apoptosis effects (sub G1) and cell cycle on FC, MFD and FD treatment. HIG-82 synoviocytes (1.5×10^5^) were plated in 60-mm cultured dishes for 24 h. The culture medium was replaced with FC, MFD, and FD media and then continued cultivating for additional 48 h. Cells were then collected, washed with PBS, fixed in PBS-methanol (1:2 v/v) solution and maintained at 4°C for at least 18 h. After one washed with PBS, the cell pellets were then stained with a PI solution containing PBS, PI (40 μg/mL), and DNase-free RNase A (40 μg/mL) for 30 min at RT in the dark. The cell pellets were then analyzed using a Becton-Dickinson FACSan flowcytometer. Data in each panel represent the percentages of sub G1, G1, S and G2/M phases.
(TIF)

S2 Fig TUNEL analysis on FC, MFD and FD treatment. HIG-82 synoviocytes (1.5×10^5^) were plated in 60-mm cultured dishes for 24 h. The culture medium was replaced with FC, MFD, and FD media and then continued cultivating for additional 48 h. Cells were fixed in 1% paraformaldehyde in PBS for 30 min, and then washed with PBS, and stored in 70% methanol at 4°C. After rehydration in PBS, cells were evaluated with TUNEL assay. Data in each panel represent the percentages of apoptosis. The values shown are mean ± SD (n = 5–8 samples per experiment). Significant differences from the FC group are *p*<0.05 (*), *p*<0.001 (**) and the MFD group are *p*<0.01 (##), respectively.
(TIF)

S3 Fig Caspase 8 analysis on FC, MFD, FD and TNF-α treatments. HIG-82 synoviocytes (1.5×10^5^) were plated in 60-mm cultured dishes for 24 h. The cultured media were replaced with FC, MFD, and FD media for 48 h or 100 ng/ml TNF-α(a positive control) for 24 h. Aliquot 1×10^5^ cells were suspended in an F-12 medium, and then homogeneous FITC-IETD-FMK substrate reagent was added to the cells, maintaining a 1:1 ratio of reagent to cell solution. After 1 h of incubation at 37°C, the cells were washed once with PBS, collected by centrifugation, and suspended in PBS. The peaks represented the mean FITC fluorescence intensities in cells were analyzed using a Becton-Dickinson FACS-Calibur flow cytometer. The red line, green line, blue line and purple line represent FC, MFD, FD and TNF-αtreatments, respectively.
(TIF)

S4 Fig Caspase 9 analysis on FC, MFD, FD and epirubicin (Epi) treatments. HIG-82 synoviocytes (1.5×10^5^) were plated in 60-mm cultured dishes for 24 h. The cultured media were replaced with FC, MFD, and FD media for 48 h or 500 nM Epi (a positive control) for 24 h. Aliquot 1×10^5^ cells were suspended in an F-12 medium, and then homogeneous FITC-LEHD-FMK substrate reagent was added to the cells, maintaining a 1:1 ratio of reagent to cell solution. After 1 h of incubation at 37°C, the cells were washed once with PBS, collected by centrifugation, and suspended in PBS. The peaks represented the mean FITC fluorescence intensities in cells were analyzed using a Becton-Dickinson FACS-Calibur flow cytometer. The red line, green line, blue line and purple line represent FC, MFD, FD and epirubicin (Epi) treatments, respectively.
(TIF)

S1 Table Folate concentrations in cells and media. (TIF)

## References

[1] Blanco FJ, Lopez-Armada MJ, Maneiro E. Mitochondrial dysfunction in osteoarthritis. Mitochondrion. 2004; 4: 715–728. 16120427 10.1016/j.mito.2004.07.022

[2] Li D, Xie G, Wang W. Reactive oxygen species: the 2-edged sword of osteoarthritis. Am J Med Sci. 2012; 344: 486–490. 22885622 10.1097/MAJ.0b013e3182579dc6

[3] Buckwalter JA, Anderson DD, Brown TD, Tochigi Y, Martin JA. The Roles of Mechanical Stresses in the Pathogenesis of Osteoarthritis: Implications for Treatment of Joint Injuries. 2013; Cartilage 4: 286–294. 25067995 10.1177/1947603513495889 PMC4109888

[4] Fox JT, Stover PJ. Folate-mediated one-carbon metabolism. Vitam Horm. 2008; 79: 1–44. 10.1016/S0083-6729(08)00401-9 18804690

[5] Stover PJ. Physiology of folate and vitamin B12 in health and disease. Nutr Rev. 2004; 62: S3–12; discussion S13. 15298442 10.1111/j.1753-4887.2004.tb00070.x

[6] Novakovic P, Stempak JM, Sohn KJ, Kim YI. Effects of folate deficiency on gene expression in the apoptosis and cancer pathways in colon cancer cells. Carcinogenesis. 2006; 27: 916–924. 16361273 10.1093/carcin/bgi312

[7] Hsu HC, Chiou JF, Wang YH, Chen CH, Mau SY, Ho CT, et al Folate deficiency triggers an oxidative-nitrosative stress-mediated apoptotic cell death and impedes insulin biosynthesis in RINm5F pancreatic islet beta-cells: relevant to the pathogenesis of diabetes. PLoS One. 2013; 8: e77931 10.1371/journal.pone.0077931 24223745 PMC3817167

[8] Kao TT, Chu CY, Lee GH, Hsiao TH, Cheng NW, Chang NS, et al Folate deficiency-induced oxidative stress contributes to neuropathy in young and aged zebrafish—Implication in neural tube defects and Alzheimer's diseases. Neurobiol Dis.2014; 71: 234–244. 25131448 10.1016/j.nbd.2014.08.004

[9] Tjiattas L, Ortiz DO, Dhivant S, Mitton K, Rogers E, Shea TB. Folate deficiency and homocysteine induce toxicity in cultured dorsal root ganglion neurons via cytosolic calcium accumulation. Aging Cell. 2004; 3: 71–76. 15038821 10.1111/j.1474-9728.2004.00086.x

[10] Kruman II, Culmsee C, Chan SL, Kruman Y, Guo Z, Penix L, et al Homocysteine elicits a DNA damage response in neurons that promotes apoptosis and hypersensitivity to excitotoxicity. J Neurosci. 2000; 20: 6920–6926. 10995836 10.1523/JNEUROSCI.20-18-06920.2000 PMC6772815

[11] Övey İS, Naziroğlu M. Homocysteine and cytosolic GSH depletion induce apoptosis and oxidative toxicity through cytosolic calcium overload in the hippocampus of aged mice: Involvement of TRPM2 and TRPV1 channels. Neuroscience. 2015; 284: 225–233. 10.1016/j.neuroscience.2014.09.078 25305668

[12] Harrison RJ. Vitamin B12 levels in erythrocytes in anaemia due to folate deficiency. Br J Haematol. 1971; 20: 623–628. 5089952 10.1111/j.1365-2141.1971.tb00800.x

[13] Czeizel AE, Dudas I, Vereczkey A, Banhidy F. Folate deficiency and folic acid supplementation: the prevention of neural-tube defects and congenital heart defects. Nutrients. 2013; 5: 4760–4775. 10.3390/nu5114760 24284617 PMC3847759

[14] Zhao Y, Zhao B. Oxidative stress and the pathogenesis of Alzheimer's disease. Oxid Med Cell Longev. 2013; 2013: 316523 10.1155/2013/316523 23983897 PMC3745981

[15] Ueland PM, Refsum H. Plasma homocysteine, a risk factor for vascular disease: plasma levels in health, disease, and drug therapy. J Lab Clin Med. 1989; 114: 473–501. 2681479

[16] Kim YI. Folate and carcinogenesis: evidence, mechanisms, and implications. J Nutr Biochem. 1999; 10: 66–88. 15539274 10.1016/s0955-2863(98)00074-6

[17] Homocysteine Studies Collaboration. Homocysteine and risk of ischemic heart disease and stroke: a meta-analysis. JAMA. 2002; 288: 2015–2022. 12387654 10.1001/jama.288.16.2015

[18] Sen U, Basu P, Abe OA, Givvimani S, Tyagi N, Metreveli N, et al Hydrogen sulfide ameliorates hyperhomocysteinemia-associated chronic renal failure. Am J Physiol Renal Physiol. 2009; 297: F410–419. 10.1152/ajprenal.00145.2009 19474193 PMC2724247

[19] Lominadze D, Roberts AM, Tyagi N, Moshal KS, Tyagi SC. Homocysteine causes cerebrovascular leakage in mice. Am J Physiol Heart Circ Physiol. 2006; 290: H1206–1213. 16258031 10.1152/ajpheart.00376.2005 PMC2819019

[20] Lindenbaum J, Allen RH. Clinical spectrum and diagnosis of folate deficiency In: Bailey LB, editor. Folate in Health and Disease. New York 1995; pp. 43–73.

[21] Clarke R, Grimley Evans J, Schneede J, Nexo E, Bates C, Fletcher A, et al Vitamin B12 and folate deficiency in later life. Age Ageing. 2004; 33: 34–41. 14695861 10.1093/ageing/afg109

[22] Lopez HL. Nutritional interventions to prevent and treat osteoarthritis. Part II: focus on micronutrients and supportive nutraceuticals. 2012; PM R 4: S155–168. 10.1016/j.pmrj.2012.02.023 22632695

[23] Vacek TP, Kalani A, Voor MJ, Tyagi SC, Tyagi N. The role of homocysteine in bone remodeling. Clin Chem Lab Med. 2013; 51: 579–590. 10.1515/cclm-2012-0605 23449525 PMC3951268

[24] Chern CL, Huang RF, Chen YH, Cheng JT, Liu TZ. Folate deficiency-induced oxidative stress and apoptosis are mediated via homocysteine-dependent overproduction of hydrogen peroxide and enhanced activation of NF-kappaB in human Hep G2 cells. Biomed Pharmacother. 2001; 55: 434–442. 11686576 10.1016/s0753-3322(01)00095-6

[25] Yang JT, Li ZL, Wu JY, Lu FJ, Chen CH. An oxidative stress mechanism of shikonin in human glioma cells. PLoS One. 2014; 9: e94180 10.1371/journal.pone.0094180 24714453 PMC3979747

[26] Chen CH, Lin WC, Kuo CN, Lu FJ. Role of redox signaling regulation in propyl gallate-induced apoptosis of human leukemia cells. Food Chem Toxicol. 2011; 49: 494–501. 10.1016/j.fct.2010.11.031 21112369

[27] Yeh CH, Yang ST, Chen CH. *Calvatia lilacina* protein extract induces apoptosis through endoplasmic reticulum stress in human colon carcinoma cells. Process Biochem. 2011; 46: 1599–1606.

[28] Ong PL, Weng BC, Lu FJ, Lin ML, Chang TT, Hung RP, et al The anticancer effect of protein-extract from Bidens alba in human colorectal carcinoma SW480 cells via the reactive oxidative species- and glutathione depletion-dependent apoptosis. Food Chem Toxicol. 2008; 46: 1535–1547. 10.1016/j.fct.2007.12.015 18226850

[29] Hsieh TJ, Liu TZ, Lu FJ, Hsieh PY, Chen CH. Actinodaphnine induces apoptosis through increased nitric oxide, reactive oxygen species and down-regulation of NF-kB signaling in human hepatoma Mahlavu cells. Food Chem Toxicol. 2006; 44: 344–354. 16168547 10.1016/j.fct.2005.08.005

[30] Lebiedzińska A, Dabrowska M, Szefer P, Marszałł M. High-performance liquid chromatography method for the determination of folic acid in fortified food products. Toxicol Mech Method. 2008;18, 463–467. 10.1080/15376510701623870 PMC2728762 19696945

[31] Shah D, Mahajan N, Sah S, Nath SK, Paudyal B. Oxidative stress and its biomarkers in systemic lupus erythematosus. J Biomed Sci. 2014; 21:23 10.1186/1423-0127-21-23 24636579 PMC3995422

[32] Furukawa S, Fujita T, Shimabukuro M, Iwaki M, Yamada Y, Nakajima Y, et al Increased oxidative stress in obesity and its impact on metabolic syndrome. J Clin Invest. 2004; 114: 1752–1761. 15599400 10.1172/JCI21625 PMC535065

[33] Ding X, Zhang Y, Huang Y, Liu S, Lu H, Sun T. Cadherin-11 involves in synovitis and increases the migratory and invasive capacity of fibroblast-like synoviocytes of osteoarthritis. Int Immunopharmacol. 2015; 26: 153–161. 10.1016/j.intimp.2015.03.024 25824718

[34] Yang CR, Shih KS, Liou JP, Wu YW, Hsieh IN, Lee HY, et al Denbinobin upregulates miR-146a expression and attenuates IL-1β-induced upregulation of ICAM-1 and VCAM-1 expressions in osteoarthritis fibroblast-like synoviocytes. J Mol Med. 2014; 92: 1147–1158. 25052989 10.1007/s00109-014-1192-8

[35] Iwanaga T, Shikichi M, Kitamura H, Yanase H, Nozawa-Inoue K. Morphology and functional roles of synoviocytes in the joint. Arch Histol Cytol. 2000; 63: 17–31. 10770586 10.1679/aohc.63.17

[36] Georgescu HI, Mendelow D, Evans CH. HIG-82: an established cell line from rabbit periarticular soft tissue, which retains the "activatable" phenotype. In Vitro Cell Dev Biol. 1988; 24: 1015–1122. 2846503 10.1007/BF02620875

[37] Crider KS, Yang TP, Berry RJ, Bailey LB. Folate and DNA methylation: a review of molecular mechanisms and the evidence for folate's role. Adv Nutr. 2012; 3: 21–38. 10.3945/an.111.000992 22332098 PMC3262611

[38] Cutolo M, Sulli A, Pizzorni C, Seriolo B, Straub RH. Anti-inflammatory mechanisms of methotrexate in rheumatoid arthritis. 2001; Ann Rheum Dis 60:729–735. 11454634 10.1136/ard.60.8.729 PMC1753808

[39] Yu DH, Yi JK, Yuh HS, Park S, Kim HJ, Bae KB, et al Over-expression of extracellular superoxide dismutase in mouse synovial tissue attenuates the inflammatory arthritis. Exp Mol Med. 2012; 44: 529–535. 22718219 10.3858/emm.2012.44.9.060 PMC3465746

[40] Min C, Eddy SF, Sherr DH, Sonenshein GE. NF-kappaB and epithelial to mesenchymal transition of cancer. J Cell Biochem. 2008; 104: 733–744. 10.1002/jcb.21695 18253935

[41] Wen SW, Zhou J, Yang Q, Fraser W, Olatunbosun O, Walker M. Maternal exposure to folic acid antagonists and placenta-mediated adverse pregnancy outcomes. CMAJ. 2008; 179: 1263–1268. 10.1503/cmaj.080859 19047607 PMC2585135

[42] Ralph SJ, Moreno-Sanchez R, Neuzil J, Rodriguez-Enriquez S. Inhibitors of succinate: quinone reductase/Complex II regulate production of mitochondrial reactive oxygen species and protect normal cells from ischemic damage but induce specific cancer cell death. Pharm Res. 2011; 28: 2695–2730. 10.1007/s11095-011-0566-7 21863476

[43] Hwang SY, Siow YL, Au-Yeung KK, House J, O K. Folic acid supplementation inhibits NADPH oxidase-mediated superoxide anion production in the kidney. Am J Physiol Renal Physiol. 2011; 300: F189–198. 10.1152/ajprenal.00272.2010 20980407

[44] Rogers EJ, Chen S, Chan A. Folate deficiency and plasma homocysteine during increased oxidative stress. N Engl J Med. 2007; 357: 421–422. 17652662 10.1056/NEJMc066569

[45] Waypa GB, Marks JD, Mack MM, Boriboun C, Mungai PT, Schumacker PT. Mitochondrial reactive oxygen species trigger calcium increases during hypoxia in pulmonary arterial myocytes. Circ Res. 2002; 91: 719–726. 12386149 10.1161/01.res.0000036751.04896.f1

[46] Pinton P, Giorgi C, Siviero R, Zecchini E, Rizzuto R. Calcium and apoptosis: ER-mitochondria Ca^2+^ transfer in the control of apoptosis. Oncogene. 2008; 27: 6407–6418. 10.1038/onc.2008.308 18955969 PMC2844952

[47] Harr MW, Distelhorst CW. Apoptosis and autophagy: decoding calcium signals that mediate life or death. Cold Spring Harb Perspect Biol. 2010; 2: a005579 10.1101/cshperspect.a005579 20826549 PMC2944358

