# Supplementary figures and images for: Folate Deficiency Triggered Apoptosis of Synoviocytes: Role of Overproduction of Reactive Oxygen Species Generated via NADPH Oxidase/Mitochondrial Complex II and Calcium Perturbation
Source: PLoS One. 2016 Jan 15;11(1):e0146440. doi: 10.1371/journal.pone.0146440 (PMC4714898; doi:10.1371/journal.pone.0146440)

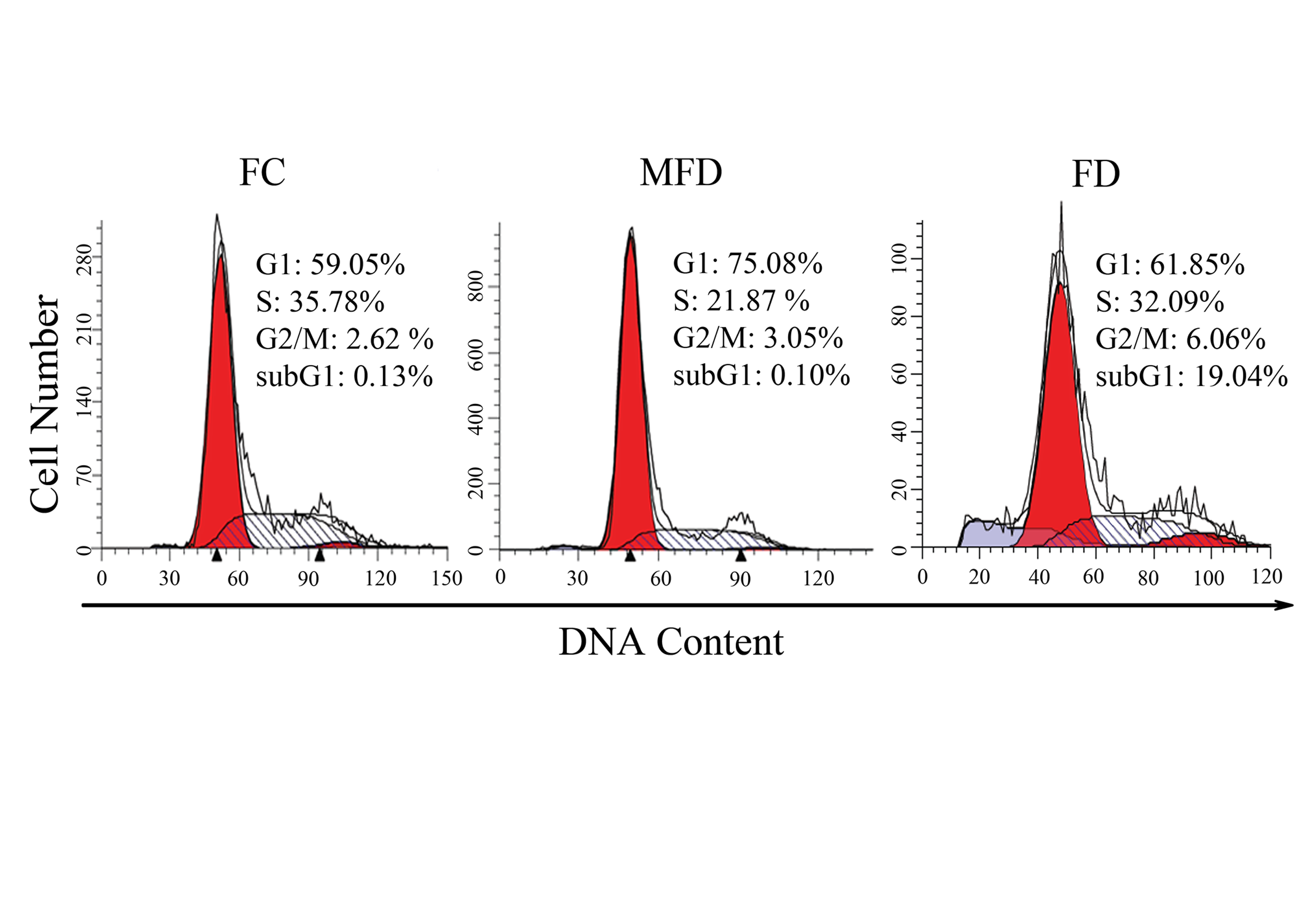

Supplement: S1 Fig — HIG-82 synoviocytes (1.5×10^5^) were plated in 60-mm cultured dishes for 24 h. The culture medium was replaced with FC, MFD, and FD media and then continued cultivating for additional 48 h. Cells were then collected, washed with PBS, fixed in PBS-methanol (1:2 v/v) solution and maintained at 4°C for at least 18 h. After one washed with PBS, the cell pellets were then stained with a PI solution containing PBS, PI (40 μg/mL), and DNase-free RNase A (40 μg/mL) for 30 min at RT in the dark. The cell pellets were then analyzed using a Becton-Dickinson FACSan flowcytometer. Data in each panel represent the percentages of sub G1, G1, S and G2/M phases. (TIF) [file pone.0146440.s001.tif]

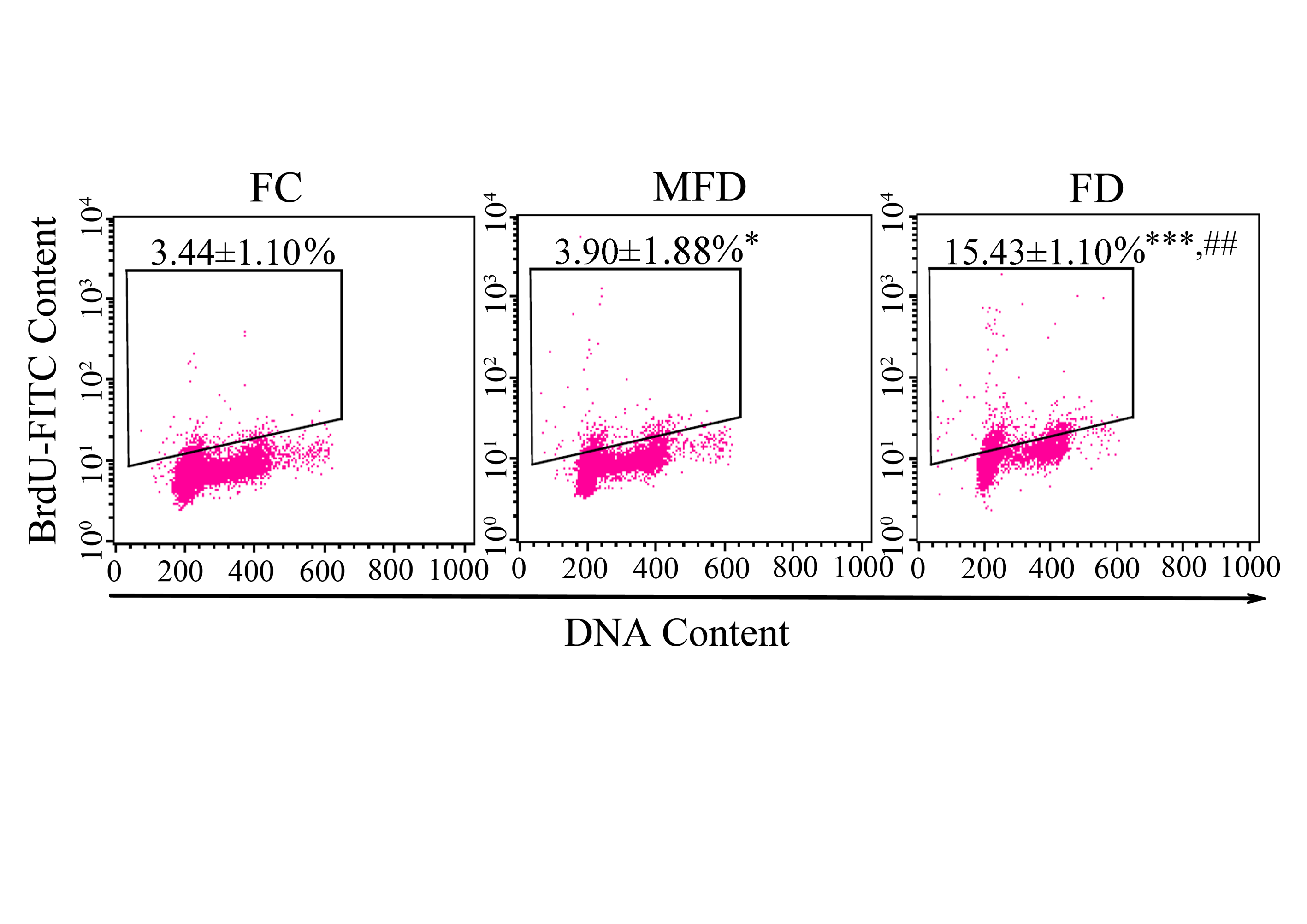

Supplement: S2 Fig — HIG-82 synoviocytes (1.5×10^5^) were plated in 60-mm cultured dishes for 24 h. The culture medium was replaced with FC, MFD, and FD media and then continued cultivating for additional 48 h. Cells were fixed in 1% paraformaldehyde in PBS for 30 min, and then washed with PBS, and stored in 70% methanol at 4°C. After rehydration in PBS, cells were evaluated with TUNEL assay. Data in each panel represent the percentages of apoptosis. The values shown are mean ± SD (n = 5–8 samples per experiment). Significant differences from the FC group are p<0.05 (*), p<0.001 (**) and the MFD group are p<0.01 (##), respectively. (TIF) [file pone.0146440.s002.tif]

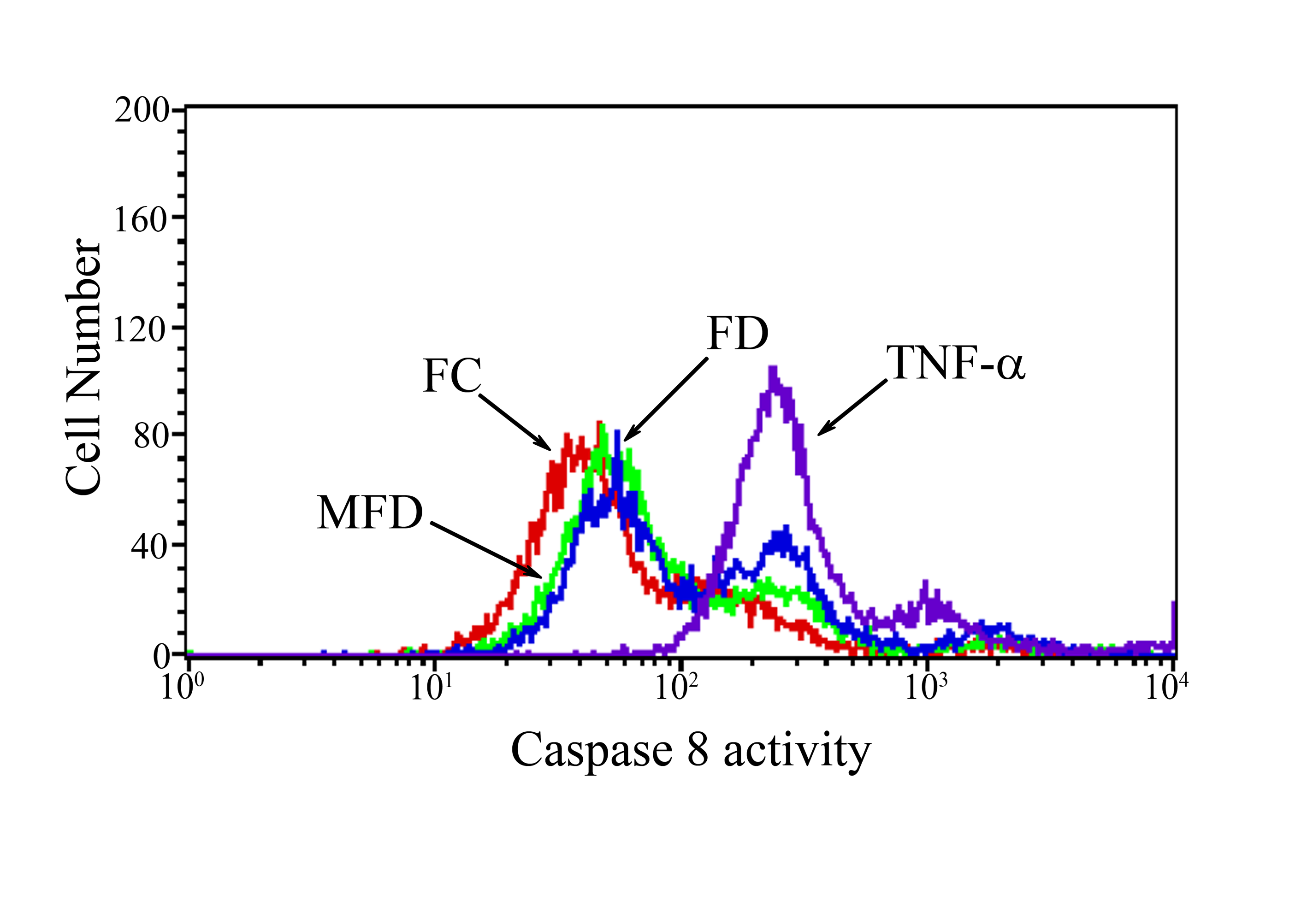

Supplement: S3 Fig — HIG-82 synoviocytes (1.5×10^5^) were plated in 60-mm cultured dishes for 24 h. The cultured media were replaced with FC, MFD, and FD media for 48 h or 100 ng/ml TNF-α(a positive control) for 24 h. Aliquot 1×10^5^ cells were suspended in an F-12 medium, and then homogeneous FITC-IETD-FMK substrate reagent was added to the cells, maintaining a 1:1 ratio of reagent to cell solution. After 1 h of incubation at 37°C, the cells were washed once with PBS, collected by centrifugation, and suspended in PBS. The peaks represented the mean FITC fluorescence intensities in cells were analyzed using a Becton-Dickinson FACS-Calibur flow cytometer. The red line, green line, blue line and purple line represent FC, MFD, FD and TNF-αtreatments, respectively. (TIF) [file pone.0146440.s003.tif]

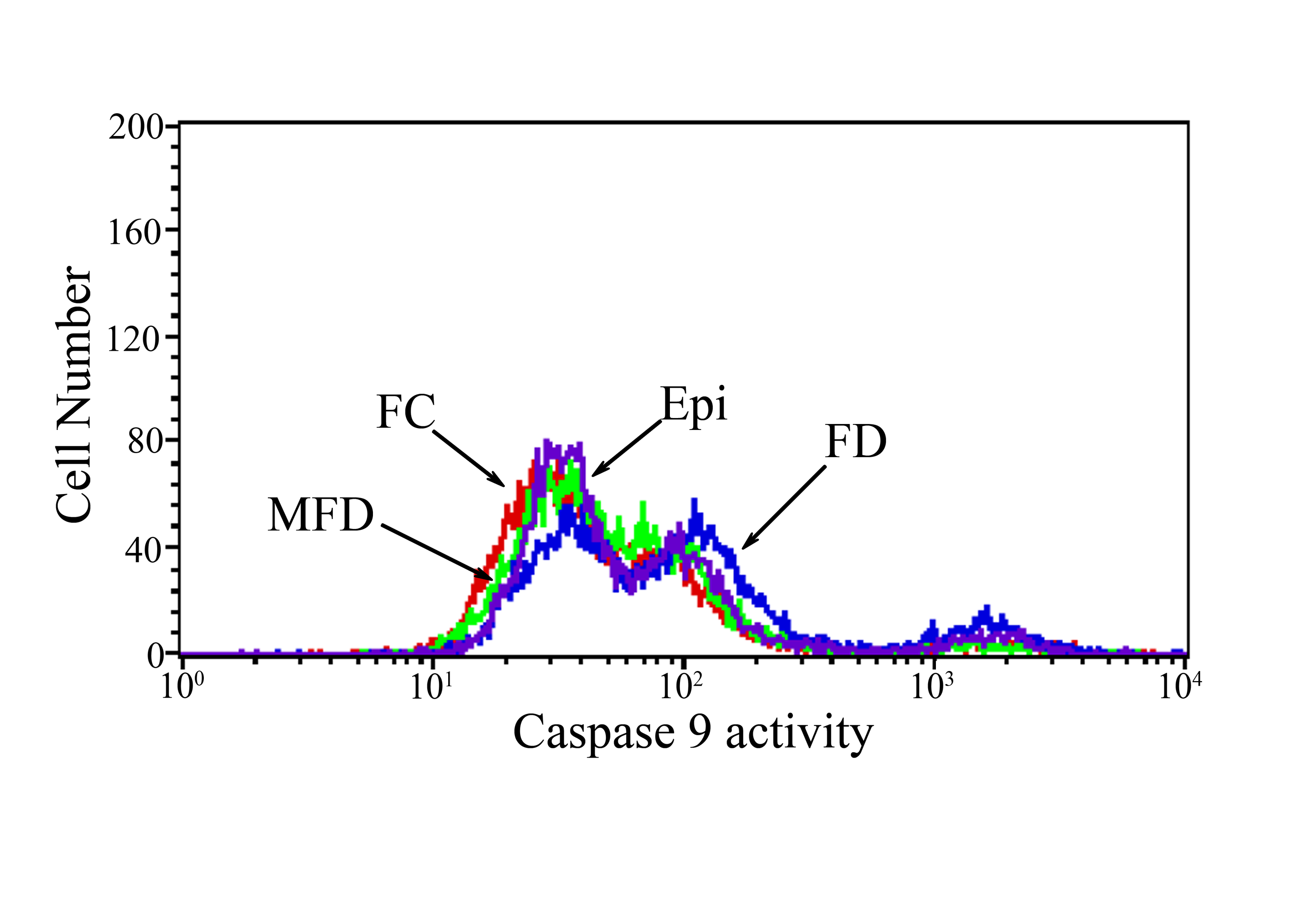

Supplement: S4 Fig — HIG-82 synoviocytes (1.5×10^5^) were plated in 60-mm cultured dishes for 24 h. The cultured media were replaced with FC, MFD, and FD media for 48 h or 500 nM Epi (a positive control) for 24 h. Aliquot 1×10^5^ cells were suspended in an F-12 medium, and then homogeneous FITC-LEHD-FMK substrate reagent was added to the cells, maintaining a 1:1 ratio of reagent to cell solution. After 1 h of incubation at 37°C, the cells were washed once with PBS, collected by centrifugation, and suspended in PBS. The peaks represented the mean FITC fluorescence intensities in cells were analyzed using a Becton-Dickinson FACS-Calibur flow cytometer. The red line, green line, blue line and purple line represent FC, MFD, FD and epirubicin (Epi) treatments, respectively. (TIF) [file pone.0146440.s004.tif]

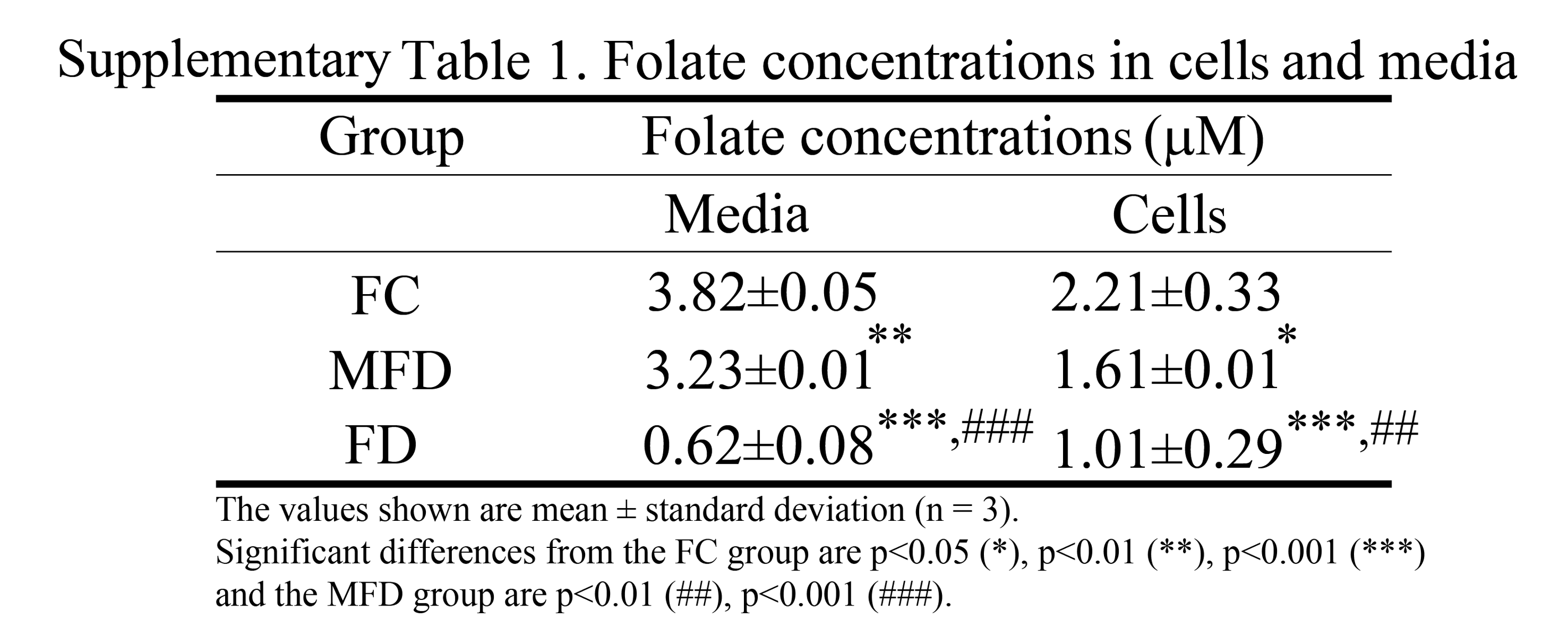

Supplement: S1 Table — (TIF) [file pone.0146440.s005.tif]
